# Genome Insights of the Plant-Growth Promoting Bacterium *Cronobacter muytjensii* JZ38 With Volatile-Mediated Antagonistic Activity Against *Phytophthora infestans*

**DOI:** 10.3389/fmicb.2020.00369

**Published:** 2020-03-11

**Authors:** Abdul Aziz Eida, Salim Bougouffa, Floriane L’Haridon, Intikhab Alam, Laure Weisskopf, Vladimir B. Bajic, Maged M. Saad, Heribert Hirt

**Affiliations:** ^1^DARWIN21, Biological and Environmental Sciences and Engineering Division, King Abdullah University of Science and Technology, Thuwal, Saudi Arabia; ^2^Computational Bioscience Research Center, King Abdullah University of Science and Technology, Thuwal, Saudi Arabia; ^3^BioScience Core Lab, King Abdullah University of Science and Technology, Thuwal, Saudi Arabia; ^4^Department of Biology, University of Fribourg, Fribourg, Switzerland; ^5^Max F. Perutz Laboratories, University of Vienna, Vienna, Austria

**Keywords:** desert bacteria, plant growth promotion, genome, abiotic stress, volatile compounds, biocontrol, *Phytophthora infestans*

## Abstract

Salinity stress is a major challenge to agricultural productivity and global food security in light of a dramatic increase of human population and climate change. Plant growth promoting bacteria can be used as an additional solution to traditional crop breeding and genetic engineering. In the present work, the induction of plant salt tolerance by the desert plant endophyte *Cronobacter* sp. JZ38 was examined on the model plant *Arabidopsis thaliana* using different inoculation methods. JZ38 promoted plant growth under salinity stress via contact and emission of volatile compounds. Based on the 16S rRNA and whole genome phylogenetic analysis, fatty acid analysis and phenotypic identification, JZ38 was identified as *Cronobacter muytjensii* and clearly separated and differentiated from the pathogenic *C. sakazakii*. Full genome sequencing showed that JZ38 is composed of one chromosome and two plasmids. Bioinformatic analysis and bioassays revealed that JZ38 can grow under a range of abiotic stresses. JZ38 interaction with plants is correlated with an extensive set of genes involved in chemotaxis and motility. The presence of genes for plant nutrient acquisition and phytohormone production could explain the ability of JZ38 to colonize plants and sustain plant growth under stress conditions. Gas chromatography–mass spectrometry analysis of volatiles produced by JZ38 revealed the emission of indole and different sulfur volatile compounds that may play a role in contactless plant growth promotion and antagonistic activity against pathogenic microbes. Indeed, JZ38 was able to inhibit the growth of two strains of the phytopathogenic oomycete *Phytophthora infestans* via volatile emission. Genetic, transcriptomic and metabolomics analyses, combined with more *in vitro* assays will provide a better understanding the highlighted genes’ involvement in JZ38’s functional potential and its interaction with plants. Nevertheless, these results provide insight into the bioactivity of *C. muytjensii* JZ38 as a multi-stress tolerance promoting bacterium with a potential use in agriculture.

## Introduction

The dramatic increase in the human population and the increasingly negative effects of climate change pose a serious threat to global food security, with a number of severe social and economic problems (FAO, 2017). Abiotic stresses, including soil salinity, nutrient deficiency, drought and heat, can cause extensive losses to agricultural yields worldwide (Boyer, 1982; Bray et al., 2000). Some of these abiotic stresses also become more intense due to global warming and climate change. For example, models have predicted a significant decrease in the percentage of soil moisture and an increase in drought-experiencing surfaces on Earth by the end of the 21st century (Burke et al., 2006; Dai, 2012). Another major abiotic stress that adversely affects plant growth and crop yields is soil salinity which affects approx. 20% of irrigated land (FAO, 2008).

Biological inoculants containing beneficial microbes are emerging as part of the 2nd green revolution technology to tackle biotic and abiotic stresses in a sustainable, environmentally-friendly and chemical-free manner and as a more rapid and cost-efficient alternative to time-consuming crop breeding (Baez-Rogelio et al., 2017; de Zélicourt et al., 2018). These beneficial microbes, including plant growth promoting bacteria (PGPB), can establish symbiotic associations and protect plants against biotic stresses, such as fungal pathogens, or promote tolerance to abiotic stresses, such as salinity or drought stress (Hardoim et al., 2008; Eida et al., 2019). PGPB may serve as biofertilizers for promoting plant growth through several mechanisms, such as increasing nutrient availability by solubilization of e.g., phosphate and zinc or sequestration of e.g., iron by siderophores (Rodriguez et al., 2004; Rodríguez et al., 2006). Other mechanisms include changes in phytohormones levels, e.g., by the production of indole-acetic acid (IAA) or modulation of ethylene levels by ACC deaminases (Idris et al., 2007; Wang et al., 2016). The production of exopolysaccharides does not only function in biofilm formation but also in water retention (Ashraf et al., 2004). Additionally, bacteria can produce volatiles (e.g., volatile organic compounds/VOCs, sulfur-containing compounds, or indole) that are used to communicate with other microbes (Weisskopf et al., 2016) or to promote growth and/or stress tolerance (Bailly et al., 2014; Liu and Zhang, 2015; Ledger et al., 2016).

Desert plants offer a promising pool of endophytic microbes from which beneficial bacteria can be isolated and exploited for their plant growth promoting potential, especially for inducing tolerance to abiotic stresses experienced in deserts (Bokhari et al., 2019). For example, we previously demonstrated the ability of the endophyte *Enterobacter* sp. SA187 isolated from the nodules of *Indigofera argentea*, a legume native to the Saudi Arabian desert, to enhance the yield of alfalfa crops using saline irrigation in the field (de Zélicourt et al., 2018). Recently, a number of desert plant species native to the Jizan region in Saudi Arabia were collected resulting in the isolation of 116 bacterial root endophyte strains of these plants (Eida et al., 2018). Here, we characterize the endophytic bacterium *Cronobacter muytjensii* JZ38 from this collection displaying significant growth promoting ability of the model plant *Arabidopsis thaliana* under salinity stress conditions in contact or contactless settings. A comprehensive genome sequence analysis of JZ38, supplemented with biochemical and phenotypic characterization, provided important insights into the pathways for conferring abiotic stress tolerance and antimicrobial activity to plants, making JZ38 a good candidate for application in agriculture.

## Materials and Methods

### Plant Assays

*Arabidopsis thaliana* wild-type Columbia (Col-0) seeds were surface sterilized by shaking for 10 min in 70% ethanol + 0.05% sodium dodecyl sulfate (SDS), then washed twice with 99% ethanol and once with sterilized ddH_2_O. The seeds were then sown on square Petri dishes (12 × 12 cm) containing half-strength Murashige and Skoog Basal Salt Mixture pH 5.8, 0.9% agar (1/2 MS) (Murashige and Skoog, 1962) (M5524, Sigma Aldrich). The plates were stored in the dark for 2 days at 4°C for seed stratification and then incubated vertically (∼75° angle) in growth chambers (Percival Scientific Inc.) at 22°C with a photoperiod of 16/8 h (light/dark, 100 μmol.m^–2^.s^–1^) for germination. Five-day old seedlings (∼1.0–1.5 cm in root length) were then gently transferred to fresh 1/2MS agar plates supplemented with 100 mM NaCl as a salt stress (five seedlings/plate). For contact (in-plate) assays, seeds were sown on 1/2MS plates containing JZ38 (2 × 10^5^ CFU/mL) prior to stratification (100 μL of pure LB was added as a control) (de Zélicourt et al., 2018). For contactless assays, sterile 15 mL Falcon tube caps (VWR) were placed at the bottom of each square Petri dish before pouring the 1/2MS media. Then, 2 mL LB agar were poured into the tube caps. Once the LB agar solidified, 50 μL (5 × 10^6^ CFU) of JZ38 were spot inoculated onto the LB agar (50 μL of pure LB was used as a control). Growth assays were performed in Percival incubators located at the KAUST Greenhouse Core Labs. Both contact and contactless assays were performed with three biological replicates.

### Shoot and Root Phenotypic Measurements

The number of lateral roots was counted under a light microscope and primary root lengths (PRL) were measured using image analysis software ImageJ^1^. Lateral root density (LRD) was calculated by dividing the number of lateral roots by the primary root length. Fresh weight (FW) was measured using a sensitive analytical balance. All measurements were taken 16 days after inoculation (DAI). For dry weight (DW) measurements, plant material was dried for 2 days at 65°C.

### Growth Conditions and Genomic DNA Isolate

Isolate JZ38 was regularly grown in LB broth (Lennox L Broth Base, Invitrogen) or on LB agar plates at 28°C. Fresh, pure bacterial cultures were used for total genomic DNA extraction using Sigma’s GenElute bacterial genomic DNA kit (Sigma Aldrich) following the manufacturer’s protocol. DNA quality and quantity were assessed by 0.7% agarose gel electrophoresis (35 V, 12 h), NanoDrop 2000 (Thermo Fisher Scientific) and Qubit dsDNA BR assay kit (Thermo Fisher Scientific).

### PacBio Library Preparation and Genome Sequencing

DNA was size selected to 10 kb using the BluePippin Size-Selection System (Sage Science), following the “High-Pass^TM^ DNA Size Selection of ∼20kb SMRTbell^TM^ Templates” manual. The SMRTbell template library was prepared according to the instructions from Pacific Biosciences’s “Procedure & Checklist - 20 kb Template Preparation using BluePippin Size-Selection System” guide. The SMRT cells were run at the KAUST Bioscience Core Labs on the PacBio *RSII* (Pacific Biosciences) sequencing platform using P6-C4 chemistry. One SMRT cell was run, taking one 360 min movie and generating 63,220 reads with a mean insert read length of 12,322 bp and an estimated genome coverage of 170X.

### Genome Assembly

Raw data from PacBio’s platform was assembled into a draft assembly using the Hierarchical Genome Assembly Process v3 (HGAP3) (Chin et al., 2013) from PacBio’s SMRT Analysis pipeline v2.3.0.140936 patch 5. The assembly workflow can be broken down to three main steps: a preassembly step that mapped single pass reads to seed reads to generate consensus reads that were then quality trimmed. *De novo* assembly was done using the overlap layout consensus approach. The final step is consensus polishing using Quiver to reduce indels and base substitution using quality scores embedded in the raw data. To determine whether assembled contigs are circular, dot plots were generated using Gepard (Krumsiek et al., 2007) for detecting overlaps at the peripheries. Overlaps were collapsed and genome was closed using Minimus2 (Sommer et al., 2007). Finally, additional polishing rounds were performed using Quiver by applying quality scores from raw data to correct for indels and base substitutions where the output from one round is inputted to the next.

### Genome Annotation

Genome annotation was carried out using the Automatic Annotation of Microbial Genomes (AAMG) which is an integrated module in the in-house INDIGO-Desert v1.1 pipeline (Alam et al., 2013). Briefly, gene prediction was done using prodigal v2.6.1 (Hyatt et al., 2010). Functional annotation was done using a multitude of tools and databases. InterProScan (Jones et al., 2014) was used to assign domain information, Gene Ontology (GO) terms and KEGG pathways. Predicted genes were compared using BLAST against UniProt^2^ for generic annotations and cross-references to Cluser of Orthologous Genes (COGs). For annotation of gene function, genes were compared to KEGG database (Functional Kyoto Encyclopedia of Genes and Genomes) (Kanehisa et al., 2016). RPS-BLAST (Marchler-Bauer et al., 2002) was used to identify conserved domains and COG (Clusters of Orthologous Groups). Predicted genes were also BLAST-ed against NCBI-nr, UniProt and KEGG. Ribosomal RNAs (rRNAs), transfer RNAs (tRNAs) and other non-coding RNAs (ncRNAs) were predicted using RNAmmer 1.2 (Lagesen et al., 2007), tRNAscan-SE 2.0 (Lowe and Eddy, 1997), and Infernal software (Nawrocki and Eddy, 2013), respectively. Prophage sequences were identified, annotated and graphically displayed using PHAST (Zhou et al., 2011). Function and pathway analysis was also performed using BlastKOALA web tool of KEGG database (Kanehisa et al., 2016). Toxin-antitoxin (T/A) systems were retrieved by using TA finder (Shao et al., 2011). Identification of gene clusters responsible for the biosynthesis of secondary metabolites was performed using antiSMASH v.4.2.0 (Weber et al., 2015). Chromosome and plasmid maps were generated using DNAPlotter release 18.0.2 (Carver et al., 2008).

### Fatty Acid Analysis

The fatty analysis was done in the BCCM/LMG services, University of Gent, Belgium. In brief, isolate JZ38 and type strain *Cronobacter sakazakii* LMG 5740^T^ were grown for 24 h at 28°C under aerobic conditions on LMG medium. Inoculation and harvesting of the cells, the extraction and analysis performed conform to the recommendations of the commercial identification system MIDI (Microbial Identification System, Inc., DE, United States). The whole-cell fatty acid composition was determined by gas chromatographically on an Agilent Technologies 6890N gas chromatograph (Santa Clara, CA, United States). The peak naming table MIDI TSBA 5.0 was used. The experiment was performed once as a standard method at BCCM/LMG.

### Phylogenetic Analysis

#### 16S rRNA-Based Phylogenetic Tree

The 16S rRNA gene sequences of JZ38 were predicted using RNAmmer 1.2 and the most common and identical sequences of the six copies were compared to known sequences listed in NCBI’s GenBank using BLASTn (Altschul et al., 1997). The sequences with the highest similarity in terms of sequence identity and query coverage, along with other type-strains from similar and distant genera were used for the phylogenetic tree construction. Multiple alignment of the nucleotide sequences was performed using MUSCLE (Edgar, 2004). The phylogenetic tree was constructed by the Neighbor-Joining method (Saitou and Nei, 1987), based on the Kimura 2-parameter model (Kimura, 1980), with bootstrap analysis (1,000 replications) using the software MEGA version 7 (Kumar et al., 2016).

#### Genome-Based Phylogenetic Tree

Using NCBI’s taxonomy website, a list of representative strains (Supplementary Table S1) belonging to different species from the *Cronobacter* genus were compiled. To help root the tree, *Mangrovibacter plantisponsor* DSM 19579 was used as an out-group. To minimize bias due to differences in annotation pipelines, all genomes were re-annotated using AAMG with identical parameters while keeping all plasmids in the analysis. Using AAMG annotations, OrthoFinder (Letunic and Bork, 2016) was used with default settings to disentangle the pan genome of the selected genomes in the form of a set of orthologous groups. Briefly, an all-vs.-all BLASTp analysis (Altschul et al., 1990) was done for the initial allocation of gene pairs. The gene pairs were then screened based on the length-normalized BLAST bitscores to generate a gene pair graph for all-vs.-all species. Next, the MCL tool v14.137 (Li et al., 2003) was used to infer orthogroups from the graph.

A gene tree was generated for each orthogroup that has at least two genomes present using the alignment-free tool DendroBlast (Kelly and Maini, 2013) and FastMe v2.1.10 (Lefort et al., 2015). A species tree was then built from the consensus of all gene trees using STAG v1.0.0^3^ and rooted based on duplication events using STRIDE v1.0.0 (Emms and Kelly, 2017). Support values were calculated based on supporting consensus from the gene trees.

For distinguishing between *Cronobacter* strains at species level, Digital DNA-DNA Hybridization (dDDH) and Average Nucleotide Identity (ANI) calculations were performed. Pairwise BLAST-based Average Nucleotide Identity values (ANIb) were obtained using JSpecies (Richter et al., 2015). The genome sequence data were uploaded to the Type (Strain) Genome Server (TYGS), a free bioinformatics platform available under https://tygs.dsmz.de, for a whole genome-based taxonomic analysis (Meier-Kolthoff and Göker, 2019).

### Determination of PGP Traits and Bioassays

Phenotypic classification was performed using API 20E (Biomérieux) strips for identification of Enterobacteriaceae according to manufacturer’s instructions. Cytochrome oxidase activity was tested using oxidase strips (Sigma Aldrich). Solubilization of phosphate and zinc, production of siderophores, evaluation of growth in different NaCl and PEG concentrations and antibiotic resistance were performed as previously described by Andrés-Barrao et al. (2017) with the slight modifications. Siderophore production and phosphate solubilization were assessed using the Blue Agar CAS assay (as described by Louden et al., 2011) and Pikovskaya’s (PVK) agar plates (M520, Himedia), respectively, by spot inoculation of 10 μL of 10^7^ CFU/mL on the respective agar surface and incubated at 28°C for 2-3 days. Evaluation of growth in salinity, osmotic stress (PEG) and pH were performed in LB broth, by inoculation into 48-well plates at starting OD_600_ of 0.01, and PEG 8000 (Fisher Scientific) was used. Growth in heavy metal stress was performed in MM9 (g/L: 25.6, Na_2_HPO_4_; 6.0, KH_2_PO_4_; 1.0, NaCl; 2.0, NH_4_Cl and separately 40 mL, 20% glucose solution; 4 mL, 1 M MgSO_4_; 200 μL, 1 M CaCl_2_; 200 μL, 0.5% thiamine) supplemented with 100 mg/L of Manganese (Mn), Cadmium (Cd), Copper (Cu), Cobalt (Co), and Nickel (Ni) using MnCl_2_, CdCl_2_.2.5H_2_O, CuCl_2_.2H_2_O, CoCl_2_.6H_2_O and NiSO_4_, respectively. Carbohydrate utilization was performed using API 50 CH (Biomérieux) strips according to manufacturer’s instructions.

Indole-3-acetic acid (IAA) and indole production was quantitatively determined by inoculating 10^5^ cells in LB broth supplemented with or without 2.5 mM L-Tryptophan (Sigma Aldrich) and incubated at 28°C with shaking at 210 rpm for 24 h. Cells were centrifuged and the supernatant was used for determination of IAA (or its precursors) and indole production. For IAA quantification, Salkowski reagent (2% of 0.5 M FeCl_3_ in 35% HClO_4_ solution) was added to supernatant in 2:1 ratio followed by 30 min incubation in the dark. For indole quantification, Kovac’s reagent (Sigma Aldrich) was added to supernatant in 1:1 ratio followed by 30 min incubation in the dark. The concentration of IAA and indole produced by the bacteria was estimated using standard curve of pure IAA (Sigma Aldrich) and indole (Sigma Aldrich) and spectrophotometric measurement at 530 and 570 nm, respectively.

Production of volatile hydrogen sulfide (H_2_S) and indole were determined by using strips impregnated with lead (II) acetate [Pb (CH_3_COO)_2_] for H_2_S and Kovac’s reagent (for indole) (Sigma Aldrich) for indole. The production was tested by inoculation of culture into falcon tube containing LB broth to starting OD_600_ of 0.01 and inserting the strips on the top of the tube. Blackening of the strips was indicative of H_2_S production while color change from yellow to pink was indicative of indole production.

Motility assay was performed by spot inoculation of 10 μL of 10^7^ CFU/mL culture on LB agar plates containing 0.3% (swimming) and 0.6% (swarming) agar, and the plates were incubated at 28°C for 2 days. Antibiotic susceptibility tests were performed using antibiotic disks (BD BBL Sensi-Disc; Hardy Diagnostics HardyDisk AST) (Supplementary Table S2) and observation of halo formation to evaluate antibiotic resistance. All aforementioned bioassays were performed with at least three biological replicates.

### Electron Microscopy

Pure colonies were picked and grown in LB broth at 28°C overnight, sub-cultured the next day to OD_600_ of 0.1. Cells from the exponential phase were harvested by centrifugation at 3000 × *g*, washed twice and resuspended in 0.1 M phosphate buffer saline (PBS). Bacterial cells were then fixed with 2.5% glutaraldehyde in cacodylate buffer (0.1 M, pH 7.4) overnight and rinsed with 0.1 M cacodylate buffer. Fixed samples were post-fixed with 2% osmium tetroxide, 1.5% potassium ferrocyanide in 0.1 M cacodylate buffer for 1h and then washed with water. Samples were then dehydrated in ethanol series and embedded in Epon epoxy resin. Contrasting sections were stained with uranyl acetate and lead citrate and imaging was performed using a Titan 80-300 S/TEM (Titan Cryo Twin; FEI Company) operating at 300 kV. The sample preparation and imaging were performed at the KAUST Imaging and Characterization Core Labs.

### Gas Chromatography–Mass Spectrometry (GC/MS)

One colony from Luria-Bertani (LB) agar plate was resuspended in 3 mL of LB broth, incubated 24 h at 28°C and shaken at 190 rpm. The bacterial culture was adjusted to a density of OD_595_ = 1.0 in LB broth and inoculating 100 μL of this cell suspension were spread on LB agar medium poured into 5 cm glass Petri dishes. LB broth inoculated on LB-agar glass plates were used as controls. Isolate JZ38 was grown overnight at room temperature before collecting the volatiles during 48h using closed-loop-stripping analysis (CLSA) as described by Hunziker et al. (2015). Experiments were repeated at least three times. Trapped volatiles were extracted from the charcoal filter by rinsing the filter three times with 25 μL dichloromethane (≥99.8%, VWR). The experiment was repeated three times. The volatiles were analyzed by gas chromatography/mass spectrometry (GC/MS). The samples were injected in a HP6890 gas chromatography connected to a HP5973 mass selective detector fitted with an HP-5 ms fused silica capillary column (30 m; 0.25-mm inside diameter; 0.25-μm film; Agilent Technologies). Conditions were as follows: inlet pressure, 67 kPa; He, 15 ml/min; injection volume, 2 μL; transfer line, 300°C; injector, 250°C; electron energy, 70 eV. The gas chromatograph was programmed as follows: 5 min at 50°C, then increasing 5°C/min to 320°C and hold 1 min. The retention time and the relative abundance were determined by using OPENCHROM program (open source software) and the compounds were identified by comparison of mass spectra to database spectra (Wiley 275 mass spectral library).

### Volatile-Mediated Effects of JZ38 on *Phytophthora infestans* and Other Phytopathogenic Fungi

Split Petri dishes were used to analyze the volatile-mediated effect of JZ38 on two *Phytophthora infestans* strains (88069 and Rec01). LB agar was poured in one half and V8 agar in the other half. A plug of mycelium (5 mm) was inoculated on the V8 medium and five drops of 10 μl (5 × 10^6^ CFU) of overnight bacterial culture were spot inoculated on the LB medium (bacteria-free LB drops were used for control plates) on the same day. Plates were sealed with Parafilm, incubated at 23°C in the dark and photographed at different time points (10 days for 88069, 13 days for Rec01) from below with a reflex camera mounted on a stand. The obtained pictures were analyzed with the digital imaging software ImageJ. The mycelium area was determined with the freehand area measurement tool of ImageJ. This growth value was then compared to that measured in control plates (targets exposed to LB only), and a percentage was calculated. These dual culture assays were performed with four biological replicates (Petri dishes). This is a protocol modified from Hunziker et al. (2015). For the other phytopathogens, experiments were performed as described earlier, however, area of mycelial growth was determined 4 days after inoculation for the fungi (*Rhizoctonia solani*, *Fusarium culmorum*, and *Botrytis cinerea*) and 11 days after inoculation for the oomycete (*P. infestans* Rec01).

### Data Availability

The data for the bacterial genome assembly and sequencing was deposited in NCBI/DDBJ/EMBL database under the accession numbers CP017662, CP017663 and CP017664, biosample number SAMN05828177. The annotations obtained by in-house INDIGO pipeline are available through the KAUST library repository^4^.

### Statistical Analysis

The data were subjected to non-parametric one-way ANOVA, or Kruskal–Wallis *H* test (Kruskal and Wallis, 1952). Data were expressed as the mean ± standard error of the mean (SEM). The differences among the various treatment means were compared and the values with a *p-*value of ≤0.05 were considered statistically significant. Statistical analysis was done using DEVELVE statistical software^5^.

## Results and Discussion

### JZ38 Promotes Arabidopsis Growth Under Salinity Stress

**FIGURE 1:**
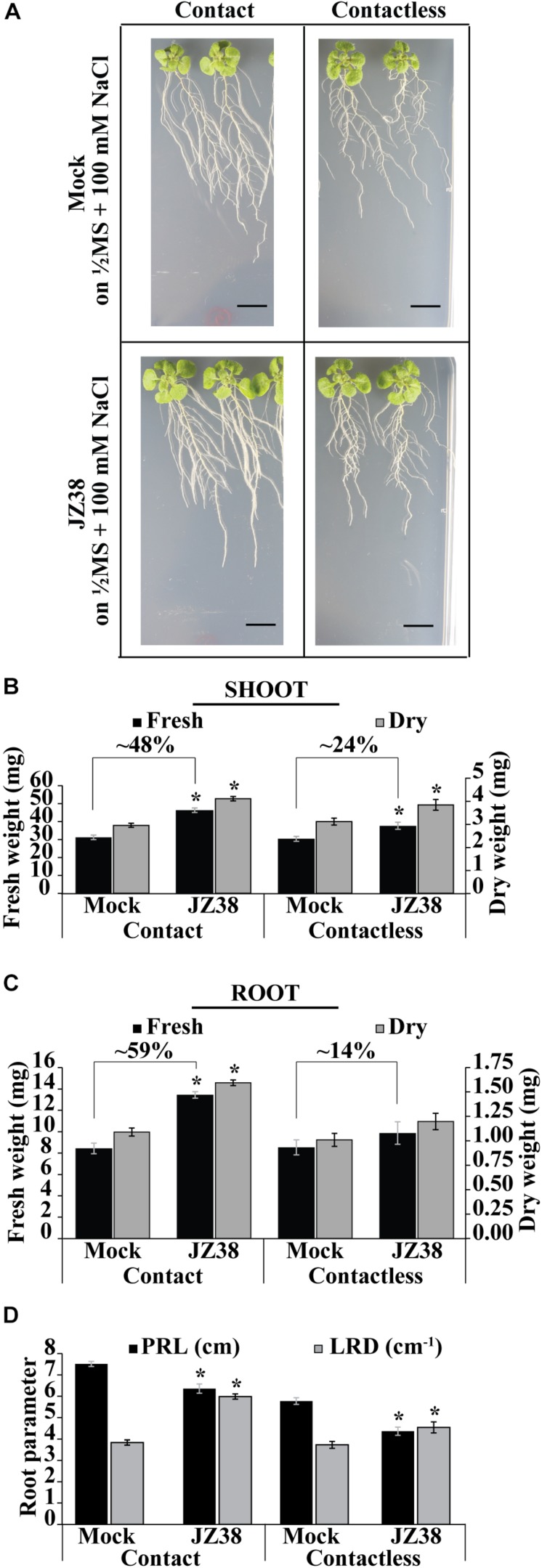
Effects of JZ38 on Arabidopsis growth under salinity stress using different inoculation methods. Representative images of *A. thaliana* plants inoculated with JZ38 or mock (bacteria-free LB) growing on 1/2MS + 100 mM NaCl, collected at 16 DAI (days after inoculation) **(A)**. Fresh and dry weight measurements of shoots **(B)** and roots **(C)** and different root parameters (PRL, primary root length; LRD, lateral root density) collected at 16 DAI **(D)**. Data are means of 3 biological replicates of 10 plants per treatment. Error bars represent standard error of the mean (SEM). Asterisks indicate significant differences between mock and bacteria-inoculated plants (Kruskal–Wallis test, *p* ≤ 0.05). Black bars correspond to 1 cm.

Previously, JZ38 was highlighted as a plant growth promoting bacteria from a collection isolated from the root endosphere of the desert plant *Tribulus terrestris* (Eida et al., 2018). Here, the exhibited phenotype and salinity stress tolerance promoting (SSTP) ability of JZ38 was quantitatively confirmed on *Arabidopsis thaliana* as a model plant. Growth promotion of plants under salinity stress (100 mM NaCl) was observed using two different inoculation methods: contact (bacteria inoculated in-plate during germination) and contactless (bacteria grown on LB caps without contact to with the plants) (Figure 1 and Supplementary Table S3). The contactless assay can be used to study the effects of volatile compounds on plant biomass and root systems at late stages of growth (Supplementary Figure S1) when compared to circular Petri dish set-ups.

Under contact setting, JZ38 increased the fresh weight of shoot (Figure 1B) and roots (Figure 1C) by 48 and 59%, respectively (Supplementary Table S3). Under contactless setting, JZ38-mediated volatiles resulted in an increase of 24% in shoot fresh weight but no statistically significant effect on root fresh weight was observed. The dry weight measurements also exhibited similar results. JZ38 also induced changes in the root system architecture by reducing primary root growth (shorter primary root length) and increasing the lateral root density of plants under salinity stress using both inoculation settings. Previously isolated endophytes of the Enterobacteriaceae family (SA187, JZ29) also exhibited a similar pattern of growth promotion with higher LRDs compared to mock plants (de Zélicourt et al., 2018; Eida et al., 2019). The ability of JZ38 to promote growth in the contactless set-up suggested that volatile compounds are emitted by JZ38 which help plants to grow under saline conditions.

### *Cronobacter* sp. JZ38 Genome Features

**FIGURE 2:**
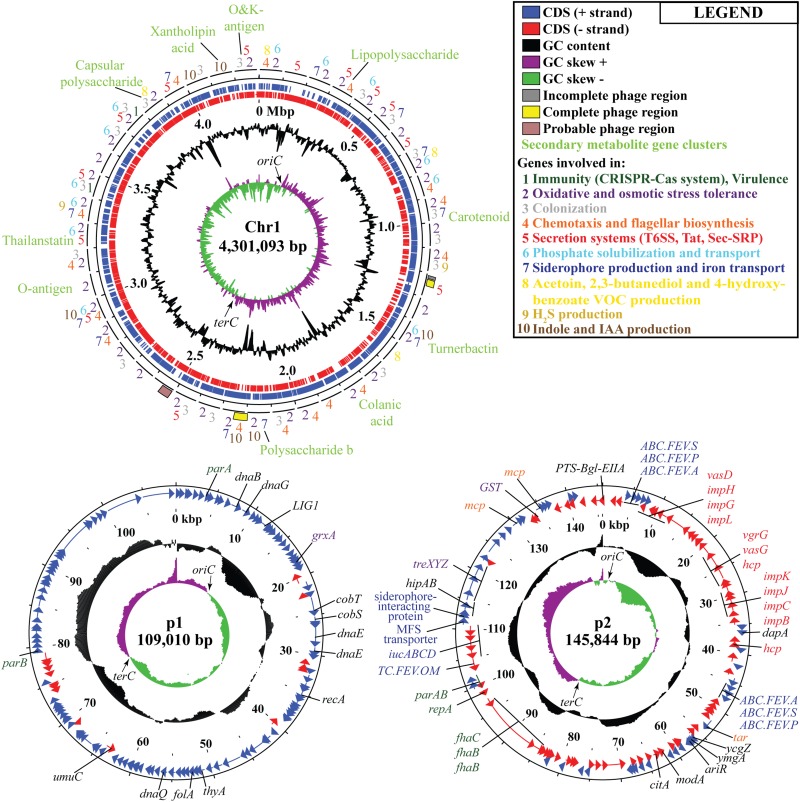
Circular representation of chromosome Chr1 and plasmids p1 and p2 in JZ38. The tracks from inside to outside: GC skew [(G-C/(G + C)] positive (**purple**) and negative (**green**),% GC content (**black**), coordinates in mega base pair (**Mbp**) for Chr1 and kilo base pair (**kbp**) for p1 and p2, reverse-strand CDSs (red), forward-strand CDSs (**blue**), phages predicted and genes present in chromosome or plasmids or the categories they belong to (**number- or color-coded according to legend**). Arrows on the GC skews indicate the origin of replication (***oriC***) and replication termination (***terC***) regions where the shifts occur. Maps generated using DNAPlotter release 18.0.2.

Following the confirmation of the plant growth promoting ability of JZ38 under salt stress conditions, whole genome sequence analysis of JZ38 was performed in order to obtain a reliable taxonomic classification and identify genes or pathways that could potentially contribute to the plant growth promoting effects. The genome of JZ38 was found to consist of three circular contigs (Figure 2): a single circular chromosome of ∼4.3 Mbp (Chr1) and two circular plasmids of ∼109 kbp (p1) and ∼145 kbp (p2) with an average GC content of 57.58, 49.04, and 58.15%, respectively (Table 1). A clear GC skew transition was observed at positions 270,964 and 2,425,765 of chromosome Chr1 corresponding to the origin of replication (*oriC*) and replication termination (*terC*) sites, respectively (Figure 2). The *oriC* and *terC* are predicted solely based on the GC skew transitions, while the *oriC* in Chr1 is confirmed by conservation of *gyrB-recF-dnaN-dnaA-rpmH-rnpA* gene organization in that region (Ogasawara et al., 1985).

**TABLE 1:** Summary of JZ38 genome features.

Feature	Chromosome 1 (Chr1)	Plasmid 1 (p1)	Plasmid 2 (p2)
Genome Size (bp)	4,301,093	109,010	145,844
DNA coding (bp)	3,766,068	96,420	127,797
DNA G + C (bp)	2,476,457	53,453	84,802
GC content (%)	57.58	49.04	58.15
G + C protein coding	2,212,283	47,934	75,809
ORF	4,260	138	125
Gene density (genes/Mbp)	990.45	1265.97	857.09
CDS	3,959	138	123
**Genes assigned to:**			
UniProt	3,932 (92.3%)	118 (85.5%)	121 (96.8%)
COG	3,353 (84.7%)	40 (33.9%)	93 (76.9%)
KEGG	3,288 (77.2%)	31 (22.5%)	88 (70.4%)
rRNAs	22		
16S-23S-5S operons	7		
5S rRNA	1		
ncRNAs	197		2
tRNAs	84		
tRNAs for standard 20 amino acids	82		
Selenocysteine tRNAs (TCA)	1		
Predicted pseudogenes	1		

A total of 4,523 ORFs were predicted in the genome of which 4,220 (3,959 in the chromosome) were putative protein-coding DNA sequences (CDS). These numbers are quite similar to those reported for other *Cronobacter* species (Chase et al., 2017; Gopinath et al., 2018; Jang et al., 2018). In addition, a total of 197 ncRNA, 7 complete rRNA operons (16S-23S-5S), one additional 5S rRNA and 84 tRNA-coding genes (82 encoding standard amino acids, 1 selenocysteine, 1 pseudogene) were predicted in the chromosome sequence, while only 2 ncRNAs were found in plasmid p2. The presence of several copies of the *rrn* operon is characteristic of soil microbes and is thought to assist in the adaptation to changing environmental or growth conditions (Klappenbach et al., 2000; Bussema et al., 2008).

### Taxonomic Affiliation of JZ38

Isolate JZ38 was first classified as *Cronobacter* sp. based on 16S rRNA gene sequence (Eida et al., 2018). Identification strip kit for Enterobacteriaceae (API 20E) phenotypically verified isolate JZ38 as a member of the genus *Cronobacter* (Supplementary Table S7). *Cronobacter* (*C.*) species, formerly known as *Enterobacter sakazakii*, are a group of gram-negative, rod-shaped bacteria that belong to the family Enterobacteriaceae (Iversen et al., 2007). The genus *Cronobacter* consists of seven species: *C. condiment*, *C. dublinensis*, *C. malonaticus*, *C. muytjensii*, *C. sakazakii*, *C. turicensis*, and *C. universalis* (Iversen et al., 2007; Iversen et al., 2008; Joseph et al., 2012).

*Cronobacter sakazakii* is an opportunistic foodborne pathogen that is classified as a public health issue due to neonatal meningitis outbreaks in powdered infant formula (Himelright et al., 2002; FAO/WHO, 2004; Drudy et al., 2006). However, there are indications that the virulence of *C. sakazakii* is due to the presence of several components found on plasmids such as pESA3 and pCTU1 (Franco et al., 2011a, b). These components include genes encoding iron acquisition systems (*eitCBAD*, *iucABCD/iutA*), type 6 secretion systems (T6SS), filamentous hemagglutinin (*fhaB*) and its transporter (*fhaC*), a RepFIB-like origin of replication (*repA*) and chromosome/plasmid partitioning proteins (*parAB*) or outer membrane serine proteases (*cpa*, plasminogen activator). A BLAST search of the sequences of the two plasmids against JZ38 revealed alignment of pESA3 (NC_009780.1) and pCTU1 (NC_013283.1) with plasmid p2 at 68 and 63% coverage, respectively, and 87% identity. Analysis of JZ38 genome revealed the presence of *iucABCD*, as part of the T6SS, two copies of *fhaB* and one copy of *fhaC, parAB*, and *repA* in plasmid p2, in addition to a complete T6SS and one copy of *fhaB* in the chromosome (Supplementary Table S6). However, the *eitCBAD* operon and the *cpa* gene were absent.

The fatty acid profiles can be used to discriminate between bacterial species (von Wintzingerode et al., 1997; Ehrhardt et al., 2010). A fatty acid profile was therefore obtained from JZ38 and the type strain of *C. sakazakii* LMG 5740^T^. The C_12__:__0_, C_14__:__0_, C_16__:__0_, C_17__:__0__cyclo_, C_18__:__1_ ω7*c*, together with summed features 2 and 3 (Table 2), were the most abundant and present FAME in the two strains. C_17__:__0_ was also present in both strains, but at a lower percentage. Isolate JZ38 exhibited a similar FAME profile to *C. sakazakii*, indicating that they belong to the same genus. The presence of C_18__:__0_ in JZ38 differentiates it from *C. sakazakii*, indicating that the two strains belong to different species.

**TABLE 2:** Comparison of cellular fatty acid compositions of JZ38 and *Cronobacter sakazakii* LMG 5740^T^.

Fatty acid (%)	*C. sakazakii* LMG 5740^T^	JZ38
Summed feature 2^†^	0.87	0.49
C_12:0_	2.92	5.34
C_14:0_	9.81	6.70
Unknown	0.85	1.23
Summed feature 2^†^	14.17	10.02
Summed feature 3^†^	24.96	16.50
C_16:1_^ω^^5c^	0.30	*n.d.*
C_16:0_	20.29	26.67
C_17:0 cyclo_	3.22	5.18
C_17:0_	0.31	0.63
C_18:1_^ω^^7c^	22.30	24.17
C_18:0_	*n.d.*	2.52
C_19:0 cyclo_^ω^^8c^	*n.d.*	0.54
Summed feature 2^†^	15.03	10.51
Summed feature 3^†^	24.96	16.50

*Values are percentages of total gas chromatogram peak area. ^†^Summed features represent two or three fatty acids that cannot be separated by the Microbial Identification system; Summed feature 2 consisted of C_12:0_ alde/unkn 10.9525 and/or C_16:1_ iso I/C_14:0_ 3OH; Summed feature 3 consisted of C_16:1_^ω^^7c^ and/or C_15_ iso 2OH; n.d., not detected/identified peaks.*

**FIGURE 3:**
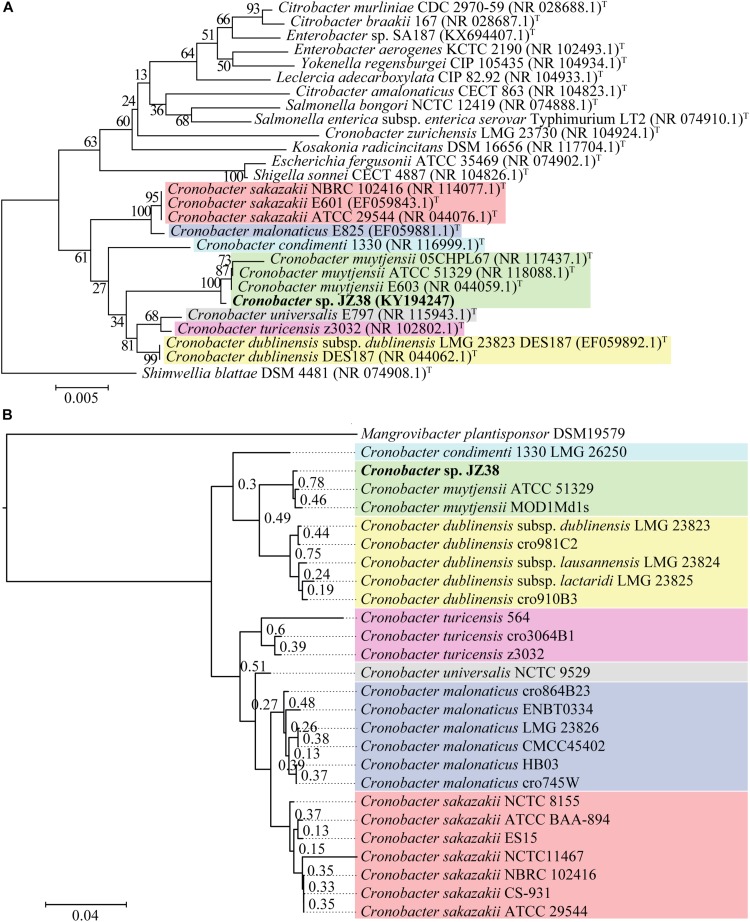
Taxonomic classification of JZ38. **(A)** 16S rRNA-based phylogenetic tree, evolutionary relationships inferred using the Neighbor-Joining method and the evolutionary distances computed using the Kimura 2-parameter method. GenBank accession numbers of isolates are presented in parentheses and type strains are indicated by a ^T^ after the parentheses. The percentage of replicate trees in which the associated taxa clustered together in the bootstrap test (1000 replicates) are shown next to the branches; **(B)** Genome-based phylogenetic tree, genomes of different species of *Cronobacter* genus were aligned using DendroBlast and FastMe and species tree was built using STAG and rooted based on duplications using STRIDE, and support values were calculated based on supporting consensus from the gene trees.

A taxonomic classification of JZ38 based on the complete 16S rRNA gene sequence (extracted from the JZ38 genome) revealed the phylogenetic affiliation of JZ38 to *C. muytjensii* (Figure 3A). To obtain a more accurate taxonomic classification and confirm the affiliation of JZ38, a whole genome based phylogenetic analysis was performed using strains related to the genus *Cronobacter* (seven different species) (Figure 3B and Supplementary Table S1). The analysis confirmed the affiliation of JZ38 with other *C. muytjensii* species and separated them from other species of this genus, such as the opportunistic food-borne pathogen *C. sakazakii*. Finally, *C. muytjensii* ATCC 51329 showed the highest similarity to JZ38 with an ANIb value of 98.74% and a dDDH value of 93.3% (formula d_6_), which are well above the species cutoff threshold of 95 and 70%, respectively, further confirming the affiliation of JZ38 to this species.

### Function Analysis of *Cronobacter muytjensii* Strain JZ38

In order to understand the metabolic capacity and plant growth promoting potential of JZ38, a functional analysis (BlastKOALA) was performed and identified 2,678 genes (67.6% of all CDSs) with assigned functions on the chromosome Chr1, while 19 (13.8%) and 53 (43.1%) genes were assigned to plasmids p1 and p2, respectively. The JZ38 genome contains a large number of metabolic genes (such as carbohydrate and amino acid metabolism), followed by environmental information processing (such as membrane transport and signal transduction) (Table 3). AntiSMASH analysis revealed the presence of 19 clusters of genes for the biosynthesis of secondary metabolites, of which only one was present on plasmid p2 while the remaining 18 were on Chr1 (Supplementary Table S4). Most interesting are the clusters with high similarity to the biosynthesis of carotenoids, colonic acid, lipopolysaccharides and aerobactin-like and enterobactin-like (turnerbactin) siderophores. Many of these compounds may be involved in establishing symbiotic associations with plants (Danese et al., 2000; Becker et al., 2005; Bible et al., 2016). In addition to secondary metabolites, JZ38 contains genes involved in oxidative and osmotic stress tolerance, colonization, chemotaxis and flagellar assembly, secretion systems, nutrient solubilization and transport, volatile compounds and phytohormone production (Figure 2).

**TABLE 3:** Summary of major functional KEGG pathways annotation of predicated genes in JZ38 using BlastKOALA.

Functional Category	# of CDS in Chr1	# of CDS in p1	# of CDS in p2
**Metabolism**			
(1) Carbohydrate metabolism	382		7
(2) Energy metabolism	163		
(3) Lipid metabolism	67		
(4) Nucleotide metabolism	101	9	
(5) Amino acid metabolism	228		5
(6) Metabolism of other amino acids	59		1
(7) Glycan biosynthesis and metabolism	50		
(8) Metabolism of cofactors and vitamins	168	5	
(9) Metabolism of terpenoids and polyketides	36		
(10) Biosynthesis of secondary metabolites	45		1
(11) Xenobiotics biodegradation and metabolism	48		3
**Genetic Information Processing**			
(12) Transcription	4		
(13) Translation	81		
(14) Folding, sorting and degradation	51		
(15) Replication and repair	84	13	
**Environmental Information Processing**			
(16) Membrane transport:			
(a) ATP-binding cassette (ABC) transporters	170		3
(b) Phosphotransferase system (PTS)	41		3
(c) Bacterial secretion system	22		6
(17) Signal transduction:			
(a) Two-component system	106		2
(b) Other	16		
**Cellular Processes**			
(18) Transport and catabolism	7		
(19) Cell growth and death	17		
(20) Cellular community:			
(a) Quorum sensing	48		
(b) Biofilm formation	74	1	7
(21) Cell motility:			
(a) Bacterial chemotaxis	21		2
(b) Flagellar assembly	37		

Identification of phages using the PHAST web tool revealed the presence of two intact phages (*Enterobacteria* phage mEp235 and *Cronobacter* phage phiES15) (Lee et al., 2012). Moreover, one incomplete and one possible region on Chr1 also detected phages, while plasmid p1 contained a phage that is highly similar to *Salmonella* phage SSU5 (Kim et al., 2012) (Supplementary Table S5). The SSU5 phage has been suggested as a possible auxiliary component of phage cocktails for biocontrol of *Salmonella* (Kim et al., 2014), which could be the case for JZ38. Bacteria have evolved Clustered Regularly Interspaced Short Palindromic Repeats (CRISPR) and CRISPR-associated (Cas) proteins (CRISPR-Cas systems) for providing adaptive immunity and acquired resistance against bacteriophages and plasmids (Darmon and Leach, 2014). Isolate JZ38 contains two operons encoding CRISPR-Cas systems, namely Type I (two copies of *cas1*, *cas2*, three copies of *cas3*), subtype I-F (*csy1234*) and “Cascade” complex systems (*casABCDE*) (Supplementary Table S6). The presence of the CRISPR system suggests its potential in protecting JZ38 from lysogenization and phage induction.

### Survival Under Abiotic Stress Conditions

Isolate JZ38 previously exhibited the ability to survive under osmotic (20% polyethylene glycol/PEG) and salinity stress (855 mM NaCl) (Eida et al., 2018). Further experiments demonstrated JZ38’s ability to grow in salt concentrations of up to 1 M NaCl (Supplementary Figure S2A), in pH varying from 4 to 9 (Supplementary Figure S2B) and in the presence of heavy metals (100 mg/L of Mn, Cd, Cu, Co, and Ni ions) (Supplementary Figure S2C). Genome mining of JZ38 revealed the presence of genes related to the production of osmoprotectants, osmoregulation and abiotic stress tolerance such as heat, acidity and oxidative stress (Supplementary Table S8).

In bacteria, one of the early responses to osmotic stress involves the uptake and accumulation of K^+^, which along with its glutamate counter-ion, may be involved in signal transduction for secondary responses (Booth and Higgins, 1990). JZ38 contains genes encoding K^+^-uptake transporters, such as the Kdp-ATPase system (*kdpABCDE*), Trk system (*trkAGE*) and Kup (*kup*), a K^+^-efflux system (*kefBCFG*) and a K^+^-channel (*kch*) (Sleator and Hill, 2002) (Supplementary Table S8). As a secondary response to high salt concentrations, bacteria accumulate high levels of osmoprotectants (or compatible solutes), such as sugars (e.g., sucrose, trehalose), amino acids (e.g., glycine betaine, proline) or polyols (e.g., glycerol, inositol), which enable bacteria to adapt to changes in osmolarity of the external environment induced by drought or salt stress (Sleator and Hill, 2002; Reina-Bueno et al., 2012; Paul, 2013). Trehalose is synthesized via five different pathways: OtsA-OtsB, TreY-TreZ, TreS, TreT and TreP pathway (Strom and Kaasen, 1993; Paul et al., 2008). In JZ38, two copies of *otsA* and a copy of *otsB* for intracellular trehalose synthesis can be found on Chr1, while genes responsible for conversion of starch to maltodextrin (*treX*), maltodextrin to maltooligosyl-trehalose (*treY*) and, then, to trehalose (*treZ*) are present on plasmid p2 (Supplementary Table S8). For trehalose homeostasis, JZ38 contains genes for trehalose catabolism, by periplasmic trehalase (two copies of *treA*) and trehalose-6-phosphate hydrolase (*treC*) to glucose, trehalose uptake transporters (*thuEFGK*, *treB*) and a transcriptional regulator (*treR*) (Supplementary Table S8) (Strom and Kaasen, 1993).

Glycine betaine is synthesized from choline via a two-step enzymatic reaction by choline dehydrogenase (BetA) and betaine aldehyde dehydrogenase (BetB), while proline is synthesized from glutamate by the action of three enzymes (ProABC) (Mandon et al., 2003). Production of glycine betaine requires the uptake of choline by BetT, ProU and ProP transporters and ABC transporter permease OpuABC (Kappes et al., 1999; Biemans-Oldehinkel and Poolman, 2003; Ly et al., 2004). In addition to choline, ProP and ProU also plays a role in the uptake of proline and in osmotic stress tolerance (10% NaCl) (Wood, 1988). The ProU complex consists of a cytoplasmic ATPase (ProV), a transmembrane subunit (ProW) and a periplasmic binding protein (ProX) (Gul and Poolman, 2013). Furthermore, PutP and ProY have also been suggested to transport proline (Wood, 1988; Liao et al., 1997). Genome mining revealed the presence of all aforementioned genes, in addition to part of the ABC transporter complex GltIJKL involved in glutamate and aspartate uptake (*gltJKL*), a transcriptional repressor of bet genes (*betI*) and extra copies of the *opuABC* system and *proP* (Supplementary Table S8).

Genes encoding enzymes with proteolytic/hydrolytic activity against oxidative stress such as two superoxide dismutases (*SOD1* and *SOD2*), hydrogen peroxide catalases (*katE* and *katG*), alkyl hydroperoxide reductases (*ahpCF*) and thiol peroxidases (*tpx*) were present (Supplementary Table S8) (Zhao and Drlica, 2014). In addition, genes for the detoxification of free radical nitric oxides by a flavohemoprotein nitric oxide dioxygenase (*hmp*), anaerobic nitrate reduction (*norRVW*), nitric oxide sensor (*nsrR*) were found (Zeidler et al., 2004). There are also five copies of glutathione S-transferases (*GST*), four glutathione peroxidases (*gpx*), a glutathione ABC transporter (*gsiABCD*), a gamma-glutamate-cysteine ligase (*gshA*), a glutathione synthetase (*gshB*), a glutathione reductase (*gor*) and hydrolase (*ggt*), glutaredoxins (*grxABCD*) and peroxiredoxins (*BCP, ahpCF*) which function in detoxification systems (e.g., reactive oxygen species/ROS detoxification) (Fones and Preston, 2012; Zhao and Drlica, 2014). Key regulators for controlling oxidative stresses include the stress response sigma factor (*rpoS*), hydrogen peroxide sensor (*oxyR*), regulators of the superoxide radical response (*soxRS*) and ferric uptake regulator (*fur*) (Supplementary Table S8) (Chiang and Schellhorn, 2012; Zhao and Drlica, 2014). There are also a number of universal stress proteins (*uspABCEFG*), some of which confer resistance to oxidative stress while others are important for motility (Nachin et al., 2005).

Polyamines, such as putrescine, cadaverine, spermidine and spermine, are also involved during oxidative, osmotic, heat and acid stress (Rhee et al., 2007; Miller-Fleming et al., 2015). JZ38 contains genes encoding polyamine transporters (*potABCD, potFGHI, potE, puuP*), for synthesis of putrescine from L-arginine (*speAB*) and L-orthinine (two copies of *speC*), its conversion to spermidine (*speE*) and *N*-acetyl spermidine (*speG*), synthesis of spermidine from *S*-adenosyl-L-methionine (*speD*), and synthesis of cadaverine from L-lysine (*cadA*) (Furuchi et al., 1991; Pistocchi et al., 1993; Kashiwagi et al., 1997; Kurihara et al., 2005) (Supplementary Table S8). In the case of pH, a number of genes found in JZ38 were previously shown to be involved in acid resistance, including antiporters (*nhaA, nhaB*, *nhaP2, chaA, yrbG*), molecular chaperones (*groEL*, *dnaK*), starvation inducible proteins (two copies of *phoH*, *dps*), ATPases (*atpABCDEFGHI*), sigma factors and regulators (*rpoS*, *ariR, nhaR*) transcriptional repressor LexA (*lexA*) and proteases (*clpXP*) (Kuhnert et al., 2004; Guazzaroni et al., 2013; Liu et al., 2016) (Supplementary Table S8). JZ38 also possesses genes encoding for resistance to heavy metals such as copper (*copA, copC, copD*, *cueO, cueR, bhsA*) and zinc/cadmium/cobalt (*zntA, zntR*), arsenate (*arsC1*) and chromate (*chrR*) (Supplementary Table S9). However, heavy metals are also important co-factors in bacteria, and JZ38 possesses several genes encoding transporters for zinc (*znuABC*, *zntB*, *zur*), nickel (*nixA, hypA, hypB*), manganese (*mntH, mntR*) and other metals (*chaA, czcD*). Indeed, JZ38 growth was completely unaffected by addition of 100 mg/L Cu^2+^ and Mn^2+^ ions.

The presence of genes for resistance to osmotic, oxidative, acidity and heavy metal stress suggests that JZ38 possesses the potential to grow and tolerate these stresses confirming the phenotypic assays (Supplementary Figure S2).

### JZ38-Plant Interaction

An endophytic lifestyle provides access to all essential nutrients for bacterial proliferation. Consequently, the genome of JZ38 contains a multitude of genes involved in the uptake, transport and metabolism of nitrogen, sulfur and carbon-based compounds. The list of genes encoding ABC transporters, Major Facilitator Superfamily (MFS) transporters and Phosphotransferase systems (PTS) is shown in Supplementary Table S10. Genes for the transport and metabolism of carbohydrates, nitrogen and sulfur are highlighted in Supplementary Tables S11–S13, respectively. For example, JZ38 contains genes encoding several sugar transporters: arabinose (*araFGH, araE*), arabinogalactan (*ganQP*, *cycB*), cellobiose (*PTS-Cel-EIIABC*), fructose (*PTS-Fru-EIIA*, *PTS-Fru-EIIB*), galacticol (*gatABC*), galactose (*galP*), glucose (*PTS-Glc-EIIABC*), glycerol (*glpF*), glycosides (*ascF/PTS-Asc-EIIB, bglF/PTS-Bgl-EIIA*), inositol (*iolT*), lactose (three copies of *lacY*), maltose/maltodextrin (*malGFEK*, two extra copies of *malK*, *malB* and *PTS-MalGlc-EIIBC, thuEFGK*), mannitol (*PTS-Mtl-EIIA*), melibiose and raffinose (*rafB*, *scrA*/*PTS-Scr-EIIBC*, *scrY*), rhamnose (*rhaSTPQ*), ribose (*rbsACB, rbsD*), sucrose (*scrY*, two copies of *scrA*/*PTS-Scr-EIIBC*), trehalose (*thuEFGK*, *treB*) and xylose (*xylFGH*).

Phenotypic characterization of sugar metabolism using API CH 50 strips demonstrated JZ38’s ability to metabolize, by fermentation or oxidation, a range of carbon sources for growth (Supplementary Table S7). These sugars included monosaccharides (L-enantiomers of arabinose and rhamnose and D-enantiomers of ribose, xylose, galactose, glucose, fructose and mannose), disaccharides (D-enantiomers of cellobiose, maltose, lactose, melibiose, saccharose/sucrose and trehalose and gentiobiose), glycosides (amygdalin, arbutin, esculin and salicin), polysaccharides (D-raffinose), polyols (glycerol, galacticol, inositol, and D-mannitol), and salts (potassium gluconate). Isolate JZ38 has the genomic potential for the metabolism of arabinose (*araBAD*) (Fritz et al., 2014), arabinogalactan (*bgaB*/*ganA*, *ganB*) (Watzlawick et al., 2016), cellulose and cellobiose (*bcsZ*, two copies of *bglX*, *bglB*, *yliI*) (Xie et al., 2007), citric acid (*citABCDEFXG, citA* in plasmid p2) (Martín et al., 2004), galactose (*lacA, galK, galT*) (Chai et al., 2012), galacticol (*gatD, gatYZ, gatR*) (Nolle et al., 2017), glucose (*pgm*, *yihX, glk*, *pgi*), glycerol (*glpK*, *glpD*) (Holmberg et al., 1990; Yang et al., 2014), glycosides (*bglB, bglA/ascB, ascG*) (Desai et al., 2010; Zangoui et al., 2015), inositol (*iolBCDEGHI*) (Morinaga et al., 2010), lactose (*lacA*, *lacZ*) (Zeng et al., 2010), maltose/matlodextrin (*malP*, *malQ*, *malS*, *malZ, glvA*) (Boos and Shuman, 1998), mannitol (*mtlD*) (Wisselink et al., 2004), melibiose and raffinose (*rafA, rafR, scrB*) (Russell et al., 1992; Hugouvieux-Cotte-Pattat and Charaoui-Boukerzaza, 2009), rhamnose (*rhaBAD*) (Schwartz et al., 1974; Rodionova et al., 2013), ribose (*rbsK, rbsD, rbsR*) (Mauzy and Hermodson, 1992; Sigrell et al., 1998), sucrose (*scrB*, two copies of *scrK, scrR*) (Hugouvieux-Cotte-Pattat and Charaoui-Boukerzaza, 2009), trehalose (two copies of *treA, treC, treR*) (Baker et al., 2018), and xylose (*xylAB*) (Shamanna and Sanderson, 1979).

Nitrogen and sulfur are essential elements in nutrients for both bacteria and plants. The genome of JZ38 lacks genes for nitrogen fixation, but contains genes encoding for ammonium (*amt*, *glnK*), urea (*urtABCDE*), nitrate and nitrite (*nrtABC*, *narK*) transport, nitrate (*narLXKGHJI, narP*) and nitrite (*nirBD*) reduction and nitrate assimilation (*nasA, nasB* annotated as *nirB* but in the same operon as *nasA*) (Supplementary Table S12). The genome also contains genes for sulfate (*cysPUWA*, one extra copy of *cysA*), taurine (*tauACB*) and alkanesulfonate (*ssuACB*) transport, metabolism of taurine (*tauD*), alkanesulfonate (*ssuDE*) and thiosulfate (*glpE*), and finally assimilatory sulfate reduction (*cysND, cysC, cysH, cysJI*) (Supplementary Table S13).

The ability of JZ38 to metabolize a diverse range of carbon sources and the presence of genes responsible for their metabolism, along with the transport and metabolism of other micronutrients confirm JZ38’s potential for a lifestyle as a plant endophyte.

A large number of genes encoding two-component systems (TCS) for rapid sensing and adjustment to changes in the external environment were present in JZ38 (Supplementary Table S14). The TCSs in JZ38 belong to the OmpR, NtrC, NarL, LytTR, CitB and chemotaxis families. The TCSs present are important for envelope stress (*cpxAB*, *baeSR*) (Batchelor et al., 2005; Leblanc et al., 2011), cell surface adhesion, biofilm formation, motility and hemolysis (*cpxAB, rstAB, tctDE*) (Otto and Silhavy, 2002; Huang et al., 2018; Taylor et al., 2019) and capsular polysaccharide synthesis (*rcsABCD*) (Cheng et al., 2010; Paczosa and Mecsas, 2016). Some were shown to be involved in carbon catabolism (*creBC*) (Godoy et al., 2016) and nitrogen metabolism (*glnGL*, *glnKR, narXL*) (Pahel et al., 1982; Nohno et al., 1989; Schreier et al., 1989). Others were involved in responses to antimicrobial peptides and acid pH (*phoPQ*) (Fields et al., 1989; Foster and Hall, 1990), osmotic stress (*kdpDE*), heavy metals (*basRS*) (Ogasawara et al., 2012) and phosphate starvation and production of acid phosphatases (*phoBR, phoPQ*) (Kier et al., 1979; Wanner, 1996).

Production and resistance to antibiotics and antimicrobial compounds play a role in suppressing soil-borne plant pathogens or enhancing the persistence of the producers in a highly competitive environment (Raaijmakers et al., 2002; Raaijmakers and Mazzola, 2012). JZ38 was also tested for its sensitivity to a range of antibiotics and possessed a number of genes involved in antibiotic resistance, antimicrobial compounds and defense mechanisms. Among the tested antibiotics, JZ38 displayed resistance to ampicillin, linezolid and penicillin G (Supplementary Table S15). Genome mining revealed the presence of genes involved in β-lactam, cationic antimicrobial peptide (CAMP), vancomycin and antifolate resistance (Supplementary Table S16). Most notably, the presence of a β-lactamase (*ampC*), a cytosolic amidase (*ampD*), an inner membrane permease (*ampG*), regulators of AmpC (*ampH*, *nagZ*), penicillin binding proteins involved in peptidoglycan biosynthesis (*mrcAB*, *pbpC*, *mrdA*, *ftsI*, *dacB*, three copies of *dacC*, *pbpG*) and multidrug efflux system (*acrAB*, *tolC*) may confer resistance to ampicillin and penicillin (Sauvage et al., 2008; Guérin et al., 2015) (Supplementary Table S16). JZ38 could also possess fungal antagonism abilities due the presence of a putative chitinase (Chernin et al., 1995).

Bacteria have evolved Toxin-Antitoxin (TA) systems composed of a toxin and its neutralizing antitoxin (Yamaguchi et al., 2011). The toxins are believed to slow down (dormancy) or suppress growth (cell death) in order to survive in rapidly changing environments or stress conditions (Gerdes et al., 1986; Yamaguchi and Inouye, 2009). TA systems have other diverse functions and roles, including ensuring persistence of plasmids during replication, virulence, antibiotic tolerance, phage defense and biofilm formation (Hayes, 2003; Sass et al., 2014; Shidore and Triplett, 2017). A number of genes related to different classes of TA systems were found in JZ38 (Supplementary Table S16), including an adenylate cyclase toxin (*cyaA*) (Cannella et al., 2017), SOS-induced toxins (*tisB*, *symE*) and their regulator (*lexA*) (Gerdes and Wagner, 2007), membrane stress phage shock proteins (*pspABCD*) (Engl et al., 2010), toxins involved in cell division (*cptAB*, *fstZ*, *merB*) (Masuda et al., 2012) cell death (*phd*, *doc*, *clpXP*) (Smith and Magnuson, 2004), promoters of persister cells (*hha*-*tomB*, *hipAB* in plasmid p2).

The presence of TCSs and the production of, or resistance to, antibiotics, antimicrobials and TA systems may enable JZ38 to sense their environment, compete with other microbes in the soil and rhizosphere and to persist in the microbial community under biotic and abiotic stress conditions.

### Chemotaxis, Motility and Colonization

Bacterial colonization of plants begins with recognition of signals from root exudates (e.g., via TCSs) and chemotactic responses toward these signals (de Weert et al., 2002; Lugtenberg et al., 2002). Bacteria can then either flow through soil water fluxes toward the roots, or they can actively induce flagellar activity enabling internalization and colonization of different parts of the plant (van der Lelie et al., 2009). The ability to move toward plant roots, adhere and colonize the surface and systematically spread within plant tissues is an important characteristic of endophytic bacteria (Hardoim et al., 2008). Motility assays revealed the ability of JZ38 to spread on 0.3% agar (swimming) but not on 0.6% (swarming) (Supplementary Figure S3A). Transmission electron photomicrograph of JZ38 also revealed the presence of peritrichous flagella (Supplementary Figure S3B).

Indeed, genome analysis revealed the presence of genes for flagellar biosynthesis, assembly and chemotaxis (Supplementary Table S17). Genes present in the genome of JZ38 which encode for structural components of the flagellar body, important regulatory factors and determinants of chemotaxis include several operons; *flgNMABCDEFGHIJKL*, *flhDC motAB cheAW fimCD tar cheRBYZ flhBAE*, *fliYZA* and *fliCDEFGHIJKLMNOPQRST* (Supplementary Table S17) (Fitzgerald et al., 2014). In addition to extra copies of *fliC* and *fimD*, some genes encoding for parts of the type IV pilus (*hofBC, hofMNOQ*) system were also present. Furthermore, genes involved in chemotaxis, such as methyl-accepting and TCS chemotaxis proteins (four copies of *tsr* and *tar*, *trg*, *aer*, nine copies of *mcp*, and *cheRBYZV*), were also present on both chromosome Chr1 and plasmid p2 (Supplementary Table S17). Along with the presence of TCSs (Supplementary Table S14), which assist in signal recognition of exudates and adaptation to the environment within the plants, these results indicate that JZ38 possesses the potential to respond to nutrients as signals and, consequently, move toward and within plant roots.

Prior to internalization and systematic invasion of plants, bacteria may attach and adhere to the surface of roots and form microcolonies and biofilms. Bacteria can form aggregates in a self-produced matrix composed of water and exopolysaccharides (EPS), such as cellulose, called biofilms (Augimeri et al., 2015), which can assist in plant colonization (Laus et al., 2005; Yaron and Römling, 2014). Some surface components of bacterial cells, such as flagella, curli fibers, type I fimbriae are involved in the formation of biofilms (Beloin et al., 2008). Bacterial biofilms can be regulated by a mechanism by which small signaling molecules called autoinducers are used for cellular communication, called quorum sensing (QS), allowing bacteria to regulate gene expression in a cell-density-dependent manner (Fazli et al., 2014; Pérez-Montaño et al., 2014). The genome of JZ38 contains genes for colonization, root surface adhesion, biofilm formation, quorum sensing, and degradation of cell walls for internalization (Supplementary Table S18).

The presence of two recombinases/integrases (*xerC*, *xerD*) suggests the ability of JZ38 to colonize the rhizosphere and root surfaces (Martínez-Granero et al., 2005). The genome encodes genes involved in mediating surface adhesion by pili formation (*ppdD, hofBC, ppdABC, hofMNOQ*) and in lipopolysaccharides, cellulose and colanic acid biosynthesis (Dörr et al., 1998; Sauvonnet et al., 2000) (Supplementary Table S18). Cellulose and colanic acid are EPSs critical for the formation of biofilms (Danese et al., 2000). JZ38 contains the Bcs operon for the biosynthesis of cellulose (*bcsABZC*, one extra *bcsA*) (Ahmad et al., 2016). JZ38 also contains a complete gene cluster for the biosynthesis and translocation of colanic acid (*wza wzb wzc wcaABCDEF gmd fcl wcaHI manCD wcaJ wzxC wcaKLM*) and a transcriptional regulator (*mcbR*) (Supplementary Tables S4, S18) (Schmid et al., 2015). JZ38 contains enzymes (*tqsA*, *luxS*) that catalyze the synthesis of the signal precursor for autoinducer-2 mediated quorum sensing which are found in a number of Enterobacteriaceae (Rezzonico et al., 2012).

The internalization of bacteria into the plant host occurs through sites of tissue damage during growth, root hairs or intact epidermis cells requiring the active secretion of cell-wall degrading enzymes (Sprent and de Faria, 1988; Morgante et al., 2005). Cellulose, hemicelluloses and pectin are the major components of the primary cell wall. The genome of JZ38 does not contain known genes encoding cellulases or hemicellulases. However, it does contain genes for the catabolism of the hexuronate D-galacturonate, the main monomer of pectin, via the isomerase pathway (*uxaABC*, two copies of *kdgK*, *kdgA*) (Kuivanen et al., 2019) (Supplementary Table S18). The genome also encodes genes involved in transport (two copies of *exuT*, *togMNAB*, *kdgT, kdgM*), transcriptional regulation (two copies of *kdgR*, *exuR*) and catabolism of other hexuronates (*uxuAB*, *kduID*, two copies of *ogl*).

Carotenoids are organic pigments produced by plants, algae and several bacteria and fungi, which might contribute to the yellow color of JZ38. The production of these pigments has been reported to be important for root colonization and fitness under abiotic stresses (Johler et al., 2010; Mohammadi et al., 2012; Bible et al., 2016). Furthermore, the carotenoid zeaxanthin is a precursor of the phyothormone abscisic acid (ABA), which is involved in abiotic stress tolerance (e.g., drought, heat, salinity and UV) and, thus, carotenoids may play a role in the growth promotion of JZ38 (Vishwakarma et al., 2017). The carotenoid biosynthesis gene cluster in Enterobacteriaceae includes six enzymes: geranylgeranyl pyrophosphate synthase (CrtE), phytoene synthase (CrtB), phytoene desaturase (CrtI), lycopene β-cyclase (CrtY), 3,(3′)-β-ionone hydroxylase (CrtZ) and zeaxanthin glucosyl transferase (CrtX), which produce zeaxanthin diglucoside from farnesyl pyrophosphate (Sedkova et al., 2005). Farnesyl pyrophosphate is produced via the isoprenoid pathway catalyzed by a number of enzymes encoded by *dxs*, *dxr*, *ispDEFGH, idi* (isopentenyl pyrophosphate isomerase) and *ispA* (farnesyl pyrophosphate synthase). JZ38 contains the carotenoid gene cluster (*crtE-idi-crtXYIBZ*), as well as the genes in the isoprenoid pathway (*dxs*, *dxr*, *ispDEFH, ispB*, *ispA*, *idi*) (Supplementary Table S18).

### Secretion Systems and Effector Proteins

Bacteria possess protein secretion systems that play important roles in biotic interactions, such as pathogenicity and colonization (Iniguez et al., 2005; Tseng et al., 2009). Compounds secreted include antimicrobial compounds, enzymes, ions, peptides, secondary metabolites, toxins, or effectors to the surrounding environment or into host cells, triggering defense responses, altering host cell’s physiology or structure or combating stresses (Green and Mecsas, 2016). The universal two-step Sec (general secretory pathway) and Tat (Twin arginine translocation) systems are ubiquitous systems responsible for the export of proteins across the plasma membrane into the periplasmic space, which can then be exported by other secretion systems (Natale et al., 2008). The T6SS is a versatile secretion system, found in many gram-negative bacteria, responsible for the export of multiple effector proteins, ions and toxins to the extracellular milieu or into the eukaryotic or prokaryotic target cells (Hood et al., 2010; Zoued et al., 2014; Wang et al., 2015). Genome analysis revealed the presence of genes encoding Sec, Tat and Type VI secretion systems (T6SS) on chromosome Chr1 (Supplementary Table S19). The presence of these secretion systems (including additional genes encoding some parts of the T6SS) may be associated with JZ38’s ability to participate in various physiological processes, including stress responses and colonization *in planta* (Weber et al., 2009; Jani and Cotter, 2010; Shidore et al., 2012; Si et al., 2017).

### Plant Growth Promoting Traits and Potential for Growth Promotion: Nutrient Acquisition

JZ38 exhibited a number of plant growth promoting traits involved in nutrient acquisition and modulating plant hormone levels that could possibly be responsible for the growth promotion. Root exudates released by plants include organic acids, which can acidify the soil and result in the solubilization of Zn or mineral P, and siderophores, which increase Fe availability through chelation (Zhang et al., 1991; Ström, 1997; Dakora and Phillips, 2002). However, bacteria also possess the ability to produce organic acids and/or siderophores and, therefore, can assist in the promotion of plant growth in field applications with crops (Sahu et al., 2018). JZ38 displayed iron (Fe) sequestration or acquisition by production of siderophores and phosphate and zinc solubilization abilities (Supplementary Figure S3C).

Phosphate (P) is an essential macronutrient for growth of all living organisms as it is a key component of nucleic acids, phospholipids and ATP. It is a major limiting factor for plant growth due to inaccessible/insoluble form in soil or rhizosphere (Holford, 1997; Eida et al., 2017). Production of organic acids by P-solubilizing microbes is well documented, and the most common form responsible for solubilization of mineral P is gluconic acid (GA) (Rodriguez and Fraga, 1999). GA is synthesized via the direct oxidation of glucose by glucose-1-dehydrogenase (*gcd*) and its co-factor pyrrolo-quinolone quinine (PQQ) (Liu et al., 1992; de Werra et al., 2009). Genome analysis of JZ38 revealed the presence of *gcd* and *pqqBCDEF* for gluconic acid production, but a lack of *ppqA*. JZ38 also contains P transporters such as the high-affinity *pstSCAB* transporter system and low-affinity P transporter (*pit*), genes involved in phosphonoacetate degradation (*phnA*) and polyP formation (*ppk*) and genes encoding phosphatases (*ppa*, two copies of *gpx-gppA*) and TCSs (*phoBR, phoU*, two copies of *phoH*) (Supplementary Table S20) (Ohtake et al., 1996; Yuan et al., 2006; Agarwal et al., 2011).

Iron (Fe) is a micronutrient that plays an important role in DNA synthesis, respiration and photosynthesis. Fe is abundant in most soils but, similar to P, inaccessible to plants due to formation of insoluble forms at neutral pH (Mori, 1999). Siderophores are Fe-specific chelating compounds that can increase Fe availability. Siderophores can be classified into several groups; hydroxymates (e.g., aerobactin, ferrichrome, ferrioxamine B), catecholates (e.g., enterobactin, bacillibactin), carboxylates (e.g., petrobactin, rhizobactin) or phenolates (e.g., pyochelin) (Boukhalfa and Crumbliss, 2002; Raymond et al., 2015). Genome analysis revealed the presence of genes involved in the biosynthesis of enterobactin (*entABCDEF*) and aerobactin (*iucABCD* located in plasmid p2) (Supplementary Tables S4, S20). There are also genes responsible for enterobactin secretion (*entS*), Fe-enterobactin and other Fe-siderophore uptake complexes (*exbBD*, *tonB*, two copies of *fepA*, *fhuEF*), enterobactin extraction (three copies of *fes*) and siderophore regulator (*ahpC*). In the rhizosphere, siderophore production by certain bacteria may deprive Fe from potential plant-pathogenic microbes and, thus, can contribute to triggering plant immunity and protection (Bakker et al., 2013; Dellagi and Aznar, 2015). The presence of efficient Fe-uptake systems can help bacteria compete for Fe in such environments, and JZ38 possesses genes encoding two ferrous iron uptake systems (*feoABC* and *efeUOB*) and a number of ABC transporters and receptor proteins and MFS transporter (Supplementary Table S21). This suggest that JZ38 is not only able to solubilize Fe but can also import and export it to the host plant.

Zinc (Zn) is another micronutrient which is important for metabolic enzyme systems and optimal plant growth and development (Brown et al., 1993). Zn deficiency is well reported in soils around the world and is a commonly occurring problem in crop plants, leading to decreases in crop yield and nutritional quality (White and Zasoski, 1999; Mumtaz et al., 2017). Similar to P and Fe, Zn is also inaccessible to plants due to its low solubility where it is found in different forms in soil (e.g., Smithsonite/ZnCO_3_) and its solubility is highly dependent on pH (higher solubility at low pH) (Martínez and Motto, 2000; Vodyanitskii, 2010). In comparison to macronutrients P (720 mg/kg) and Fe (∼1665 mg/kg), the amount of the micronutrient Zn (∼32 mg/kg) in Jizan desert soil was much lower (Eida et al., 2018). Previously, JZ38 was identified to possess ZnO and ZnCO_3_-solubilizing abilities on agar plate assays (Eida et al., 2018). Therefore, Zn solubilization by JZ38 may have been an important trait for plant growth promotion of the *T. terrestris* from which it was isolated.

Secretion of organic acids, such as 5-ketogluconic acid and pentanoic acid, by bacteria is associated with acidification of soil or medium and subsequent solubilization of ZnCO_3_ (Saravanan et al., 2007). Indeed, Zn-solubilizing bacteria belonging to different genera of the Enterobacteriaceae family have been shown to promote the growth and Zn content of cotton (Kamran et al., 2017). The genes for the production of gluconic acid from glucose (*gcd*) and subsequent conversion to 5-ketogluconic acid (*ghrB*) were present in JZ38 (Supplementary Table S20) (De Muynck et al., 2007). In addition, a high-affinity ABC transporter for Zn import (*znuABC*), its regulator (*zur*) and efflux systems for constitutive (*zitB*) and regulated (*zntAB*, *zntR*) Zn export are also present (Supplementary Table S9) (Wang D. et al., 2012). These results suggest bacteria are able to solubilize Zn, import it into their cells and/or export it to the plant host.

### Plant Growth Promoting Traits and Potential for Growth Promotion: Phytohormone and Volatile Compound Production

Plant growth promotion can be achieved by either direct interactions between the beneficial bacteria and their host and/or antagonistic activity against plant pathogenic microbes. Plant growth and abiotic stress tolerance promotion by bacteria is well established (Dimkpa et al., 2009; Glick, 2012), but the precise mechanisms are still not fully understood. However, there are numerous indications that bacterial phytohormone production, such as IAA, can play important roles (Patten and Glick, 2002; Enebe and Babalola, 2018). Production of IAA occurs through five different pathways using tryptophan (Trp) as a precursor: indole-3-acetonitrile (IAN), indole-3-acetamide (IAM), tryptophan side-chain oxidase (TSO), tryptamine (TAM) and indole-3-pyruvate (IPyA) pathways (Patten and Glick, 1996; Carreño-Lopez et al., 2000; Spaepen et al., 2007). Biochemical analysis of JZ38 revealed its ability to produce IAA (10 μg/mL), or its precursors (IAM or IPyA) in liquid culture, with a 34% (13.4 μg/mL) increase when supplemented with 2.5 mM L-Trp (Table 4). Genomic analysis of JZ38 revealed the presence of Trp biosynthesis genes (*trpABCDE*), a Trp-specific importer (*mtr*) and IAA biosynthesis genes in the IPyA pathway (*aspC, ipdC, aldA, aldB*) (Supplementary Table S22) (McClerklin et al., 2018).

**TABLE 4:** Phytohormone and volatile compound production capabilities of JZ38.

Biochemical assay	Activity
**IAA/IAM/IPyA production (μg/mL)**	
− Trp	46.6
+ Trp	61.3
**Indole production (μg/mL)**	
− Trp	10.0
+ Trp	13.4
Indole volatile production	+
H_2_S volatile production	+

*Activity represents qualitative ability forproduction. Trp, indicates the growth of bacteria in absence (−) orpresence (+) of 2.5 mM L-Tryptophan.*

Indole is an important interspecies and inter-kingdom signaling molecule and can influence a number of biological functions (Kim and Park, 2015; Lee et al., 2015). It has been shown to promote root development and growth at specific concentrations, possibly by Trp-independent or conversion of indole back to Trp by the tryptophan synthase-β subunit (TSB1 and TSB2) (Last et al., 1991; Ouyang et al., 2000; Blom et al., 2011; Bailly et al., 2014). Phenotypic analysis revealed the ability of JZ38 to produce indole (46.6 μg/mL) in liquid, with a 31.5% (61.3 μg/mL) increase when supplemented with L-Trp, and as a volatile compound (Table 4). Indeed, JZ38 possesses the genes encoding tryptophanase enzyme for conversion of Trp to indole (*tnaA*) and excretion of indole (*acrEF*) (Supplementary Table S22) (Kawamura-Sato et al., 1999; Li and Young, 2013). Endophytic bacteria from the same plant as JZ38’s host, which displayed salinity stress tolerance promotion (SSTP) in Arabidopsis but belonged to different taxa, also produced IAA and/or indole (Eida et al., 2019).

In addition to indole volatiles, SSTP endophytic bacteria from the Jizan region (Eida et al., 2019) shared a common characteristic with JZ38 to produce sulfur-containing volatile compounds, such as hydrogen sulfide (H_2_S) (Table 4). This gaseous compound has been shown to induce salinity stress tolerance in crop plants, such as alfalfa (Wang Y. et al., 2012), barley (Chen et al., 2015) and rice (Mostofa et al., 2015), and Arabidopsis (Li et al., 2014; Shi et al., 2015). Genes required for assimilatory sulfate reduction (H_2_S production) were present in JZ38 (*cysND, cysC, cysH, cysJI*) (Supplementary Table S22). In addition, genes encoding enzymes cystathionine β synthase (*CBS*) and cystathionine-γ-lyase (*CTH*) involved in other possible pathways for H_2_S production were also present (Supplementary Table S22) (Dias and Weimer, 1998; Rose et al., 2017). Bacterial production of other volatile organic compounds (VOCs), such as acetoin and 2,3-butanediol, have also been shown to promote plant growth (Ryu et al., 2003). Indeed, JZ38 produced acetoin (Supplementary Table S7) and possessed the genes for acetoin (*ilvM*, *ilvH*, three copies of *ilvB*, *budA*) and 2,3-butanediol (*budC*, *butA*) production (Supplementary Table S22) (Taghavi et al., 2010). In addition, the gene for synthesis of the VOC 4-hydroxybenzoate (*ubiC*) (Siebert et al., 1994), which is thought to be involved in antimicrobial activity and suppression of plant pathogens, was also found in JZ38 (Walker et al., 2003).

In line with the bioassays, the GC/MS profile revealed the presence of indole and 3-methylthioindole, an intermediate in the Gassman indole synthesis (Sundberg, 1984), in the volatiles emitted by JZ38 (Table 5 and Supplementary Table S23). Additionally, sulfur molecular (octasulfur, S8), was also detected and could indicate the presence of H_2_S. Some volatiles detected are involved in antagonistic activities against pathogenic microbes. For example, 2-phenylethanol has been shown to inhibit mycelial growth of the fungus *Guignardia citricarpa* and the oomycete *P. infestans* (Fialho et al., 2011; De Vrieze et al., 2015). 2-Undecanone completely inhibited the growth of the fungi *R. solani* and *Alternaria alternata*, the nematode *Caenorhabditis elegans, Panagrellus redivivus*, and *Bursaphelenchus xylophilus* flies and slight inhibition of oomycete *P. infestans* sporangiogenesis (Gu et al., 2007; Groenhagen et al., 2013; De Vrieze et al., 2015).

**TABLE 5:** Most reproducible volatile compounds of JZ38 identified by GC/MS.

Retention time (min)	Relative abundance	Compound	CAS no.
7.47	1.05E + 06 ± 3.18E + 05	2,5-Dimethylpyrazine	123-32-0
10.74	3.83E + 04 ± 9.20E + 03	2-Ethyl-5-Methylpyrazine	13360-64-0
10.78	4.36E + 04 ± 1.11E + 04	2,3,5-Trimethyl pyrazine	14667-55-1
14.51	3.08E + 05 ± 8.10E + 04	2-Phenylethanol	60-12-8
17.65	5.83E + 04 ± 2.31E + 04	Dimethyl tetrasulfide	5756-24-1
20.20	4.76E + 06 ± 1.28E + 05	1H-Indole	120-72-9
25.16	3.36E + 04 ± 1.21E + 04	2-Undecanone	112-12-9
30.11	4.94E + 04 ± 5.46E + 03	3-Methylthioindole	40015-10-9
36.47	1.87E + 05 ± 7.41E + 04	Sulfur, Mol. (S8)	10544-50-0

*The relative abundances are average of three biological repeats ± standard deviation.*

### JZ38 Inhibits the Growth of *Phytophthora infestans* via Volatile-Emission

**FIGURE 4:**
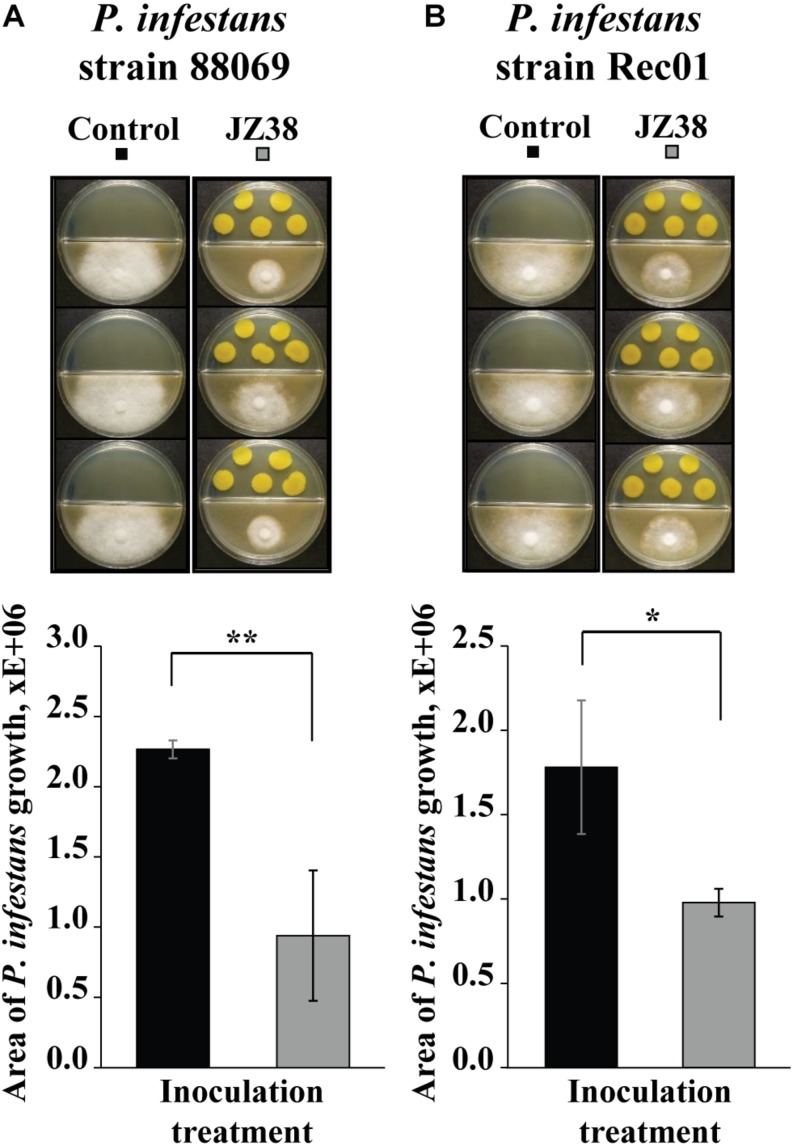
Volatile-mediated effects of JZ38 on mycelial growth of the phytopathogenic oomycete *Phytophthora infestans*. Representative images of the growth inhibition of *P. infestans* strains 88069 **(A)** and Rec1 **(B)** observed on V8 agar medium (lower side of split-dishes) mediated by volatiles emitted by JZ38 grown on LB agar medium (upper side). Area of mycelial growth was determined 10 and 13 days after inoculation for strains 88069 and Rec1, respectively. Significant differences from the LB-bacteria free control (Student’s *t* test; *n* = 3–5) are indicated by asterisks (*p < 0.05; **p < 0.01).

Since the GC/MS suggested the presence of potential antimicrobial volatile compounds, we hypothesized that JZ38 could possess volatile-mediated antagonistic activity against some phytopathogens. Therefore, the ability to inhibit the growth of two strains of the phytopathgenic oomycete *P. infestans* via volatile-emission by JZ38 was tested. As hypothesized, JZ38 exhibited growth inhibition activity against *P. infestans* via emission of volatiles (Figure 4). JZ38 inhibited the growth of strains 88069 and Rec01 by approximately 58 and 45%, respectively. However, when JZ38 was tested with phytopathogenic fungi *R. solani, F. culmorum and B. cinerea*, no antagonistic effect were observed (Supplementary Figure S4), indicating a degree of specificity by volatiles emitted by JZ38 toward phyotpathogens.

*Phytophthora infestans* is an oomycete that cause the late blight disease in potato, a major crop in Europe. The late blight disease costs approximately over 1 billion Euros annually (Haverkort et al., 2008). Some of the current solutions are synthetic chemical substances that are costly and have negative side effects on the environment and human health. The use of microbes with antagonistic activity against disease-causing pathogens (termed “biological control or biocontrol”) provides a sustainable, environmentally friendly solution to many plant diseases. Whether the use of JZ38 could suppress late blight in potato under field conditions needs to be tested. However, the potential to inhibit the growth of *P. infestans* is evident by the demonstrated plate assay.

## Conclusion

The endophytic bacterial isolate JZ38 was demonstrated to possess SSTP abilities on Arabidopsis plants either by direct contact with plant tissues or indirectly via emission of volatile compounds. Genome sequencing of the bacterium and its taxonomic analysis identified JZ38 as *Cronobacter muytjensii*, separating it from the pathogenic *C. sakazakii*. Genome analysis and additional phenotypic characterization showed that JZ38 can grow under abiotic stresses in the absence and in association with the model plant Arabidopsis. JZ38 was also demonstrated to possess genes for chemotaxis, motility, plant colonization and secretion systems. JZ38 produced a mixture of volatiles, including indole and sulfur compounds that probably underlie the contact-independent SSTP activity of JZ38. Indole might be a major player in the growth-promoting effects under salinity stress conditions and high concentrations of indole have been shown to promote shoot growth and lateral root formation and to decrease the length of the primary roots under normal conditions (Bailly et al., 2014). The high levels of indole detected in the GC/MS might explain the root phenotype observed by JZ38 inoculation in the contactless assay, suggesting a crucial role of indole in promoting salinity stress tolerance in Arabidopsis. Furthermore, the detection of VOCs with possible biocontrol activity (e.g., 2-phenylethanol and 2-undecanone) may contribute to JZ38’s VOC-mediated growth inhibition of the two oomycetes *P. infestans* strains. To our knowledge, this is the first report on the PGP potential and SSTP ability of a *C. muytjensii* strain. The results of the various assays and genome analysis reveal the potential of using JZ38 as both abiotic and biotic stress tolerance promoting bacterium in agricultural applications. However, genetic, transcriptomic and metabolomics analyses are required to confirm the highlighted genes’ involvement in JZ38’s functional potentials.

## Data Availability Statement

The datasets generated for this study can be found in the NCBI/DDBJ/EMBL database under the accession numbers CP017662, CP017663, and CP017664.

## Author Contributions

AE performed the plant screening and phenotyping assays, gDNA extraction, bioassays, and taxonomic analysis based on 16S rRNA. SB performed genome assembly, gene prediction, annotation of genome data, and taxonomic analysis based on whole genomes. IA integrated all bioinformatics databases and facilitated all genomic annotation platforms. FL’H and LW performed the volatile-mediated pathogen assays, VOCs GC/MS profile, and the data analysis. AE, MS, and HH wrote the manuscript. MS, VB, and HH conceived the overall study.

## Conflict of Interest

The authors declare that the research was conducted in the absence of any commercial or financial relationships that could be construed as a potential conflict of interest.

## Footnotes

1 https://imagej.nih.gov/ij/

2 https://www.uniprot.org/

3 https://github.com/davidemms/STAG

4 https://doi.org/10.25781/KAUST-17TW0

5 https://www.develve.net/
