# Supplementary material for: Genome Insights of the Plant-Growth Promoting Bacterium Cronobacter muytjensii JZ38 With Volatile-Mediated Antagonistic Activity Against Phytophthora infestans
Source: Front Microbiol. 2020 Mar 11;11:369. doi: 10.3389/fmicb.2020.00369 (PMC7078163; doi:10.3389/fmicb.2020.00369)
Supplement: Supplementary file 1 [file Data_Sheet_1.docx]

Supplementary Material

**
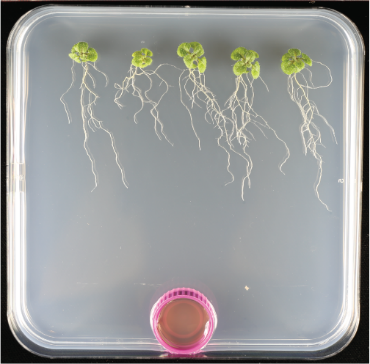
**

**Supplementary Figure S1. Example of contactless plate assay set-up used for screening effects of volatiles emitted from bacteria on Arabidopsis plants.** Bacteria are spot-inoculated on LB agar-filled Falcon tube caps (bottom, purple).

**
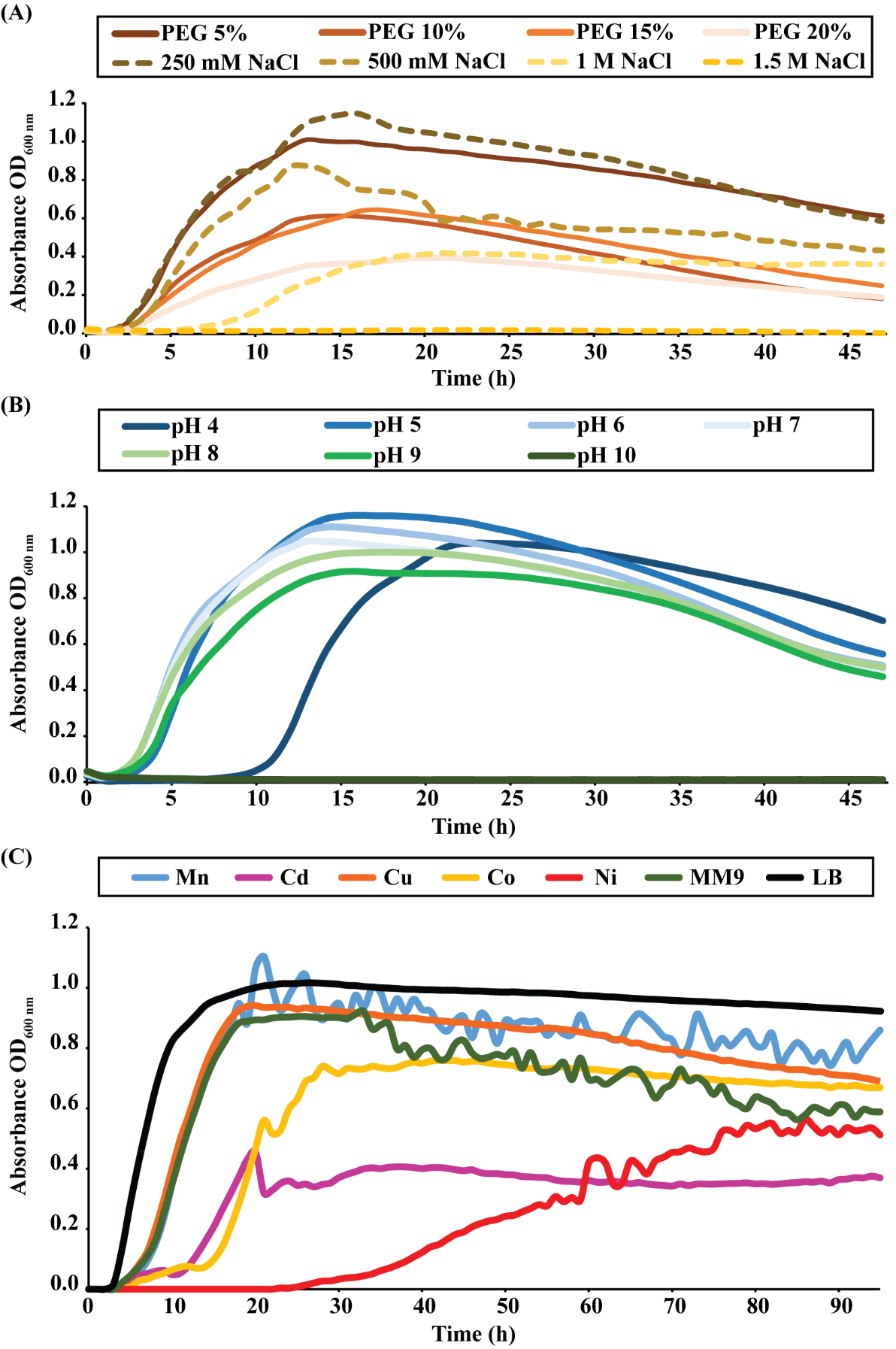
**

**Supplementary Figure S2. Growth curves of JZ38 under different abiotic stresses.** (**A**) Growth in presence of osmotic stress (PEG, solid) and salinity stress (NaCl, dotted); (**B**) Growth in acidic or basic pHs (4-10); (**C**) Growth in LB and MM9 supplemented with 100 mg/L of different metal compounds (Manganese, Mn; Cadmium, Cd; Copper, Cu; Nickel, Ni).

**
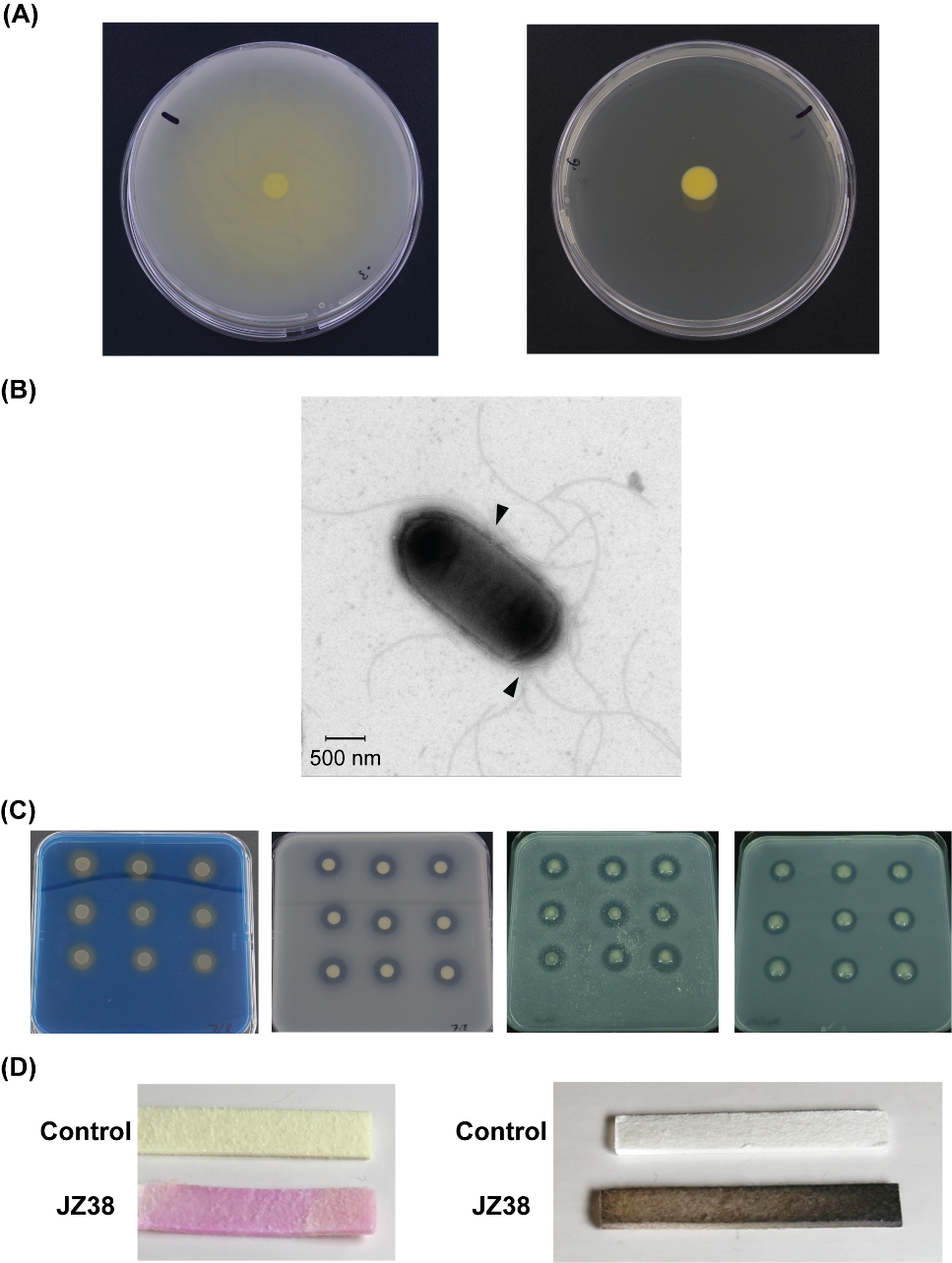
**

**Supplementary Figure S3. Qualitative analysis of JZ38’s PGP traits, abilities and properties.** (**A**) Motility assay performed by growth on 0.3% (swimming, left) and 0.6% (swarming, right) agar, mobility is exhibited by spreading of colony growth; (**B**) Transmission electron photomicrograph of JZ38. Cells were harvested during exponential phase and arrows indicate the presence of several peritrichous flagella; (**C**) Nutrient acquisition assays performed by siderophore production and solubilization of tricalcium phosphate, zinc oxide and zinc carbonate (left to right, respectively), positive ability is indicated by halo formation around colonies; (**D**) Indole (left) and hydrogen sulfide (right) production assay performed using test strips, positives exhibited by formation of pink (indole) and black (hydrogen sulfide) color.


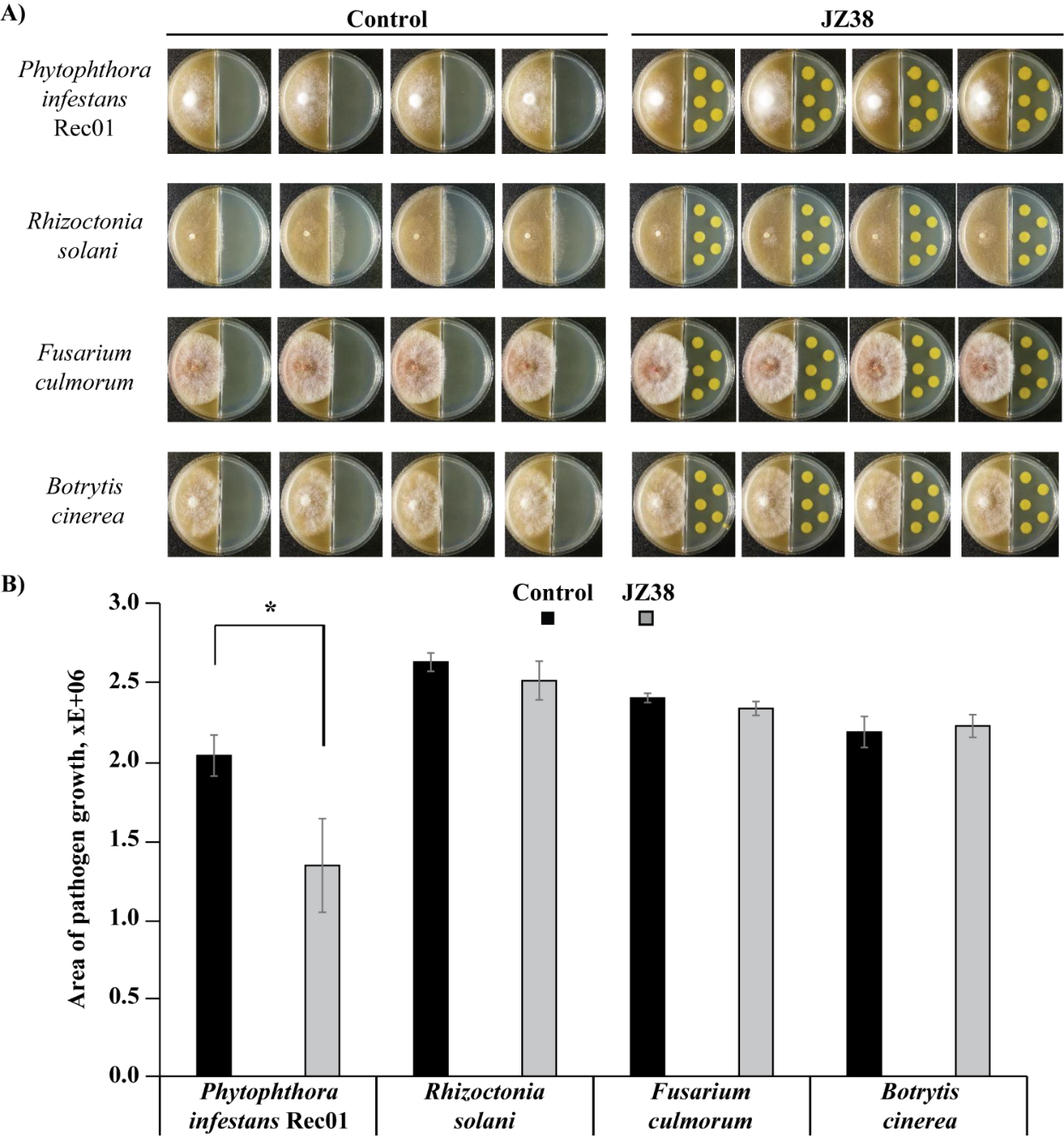


**Supplementary Figure S4. Volatile-mediated effects of JZ38 on mycelial growth of the phytopathogenic oomycete *Phytophthora infestans* Rec01 and different phytopathogenic fungi.** (**A**) Growth inhibition assays of *P. infestans* strain Rec01, *R. solani*, *F. culmorum* and *B. cinerea* on V8 agar medium (left side of split-dishes) mediated by volatiles emitted by JZ38 grown on LB agar medium (right side). (**B**) Area of mycelial growth was determined 4 days after inoculation for *R. solani*, *F. culmorum* and *B. cinerea* and 11 days after inoculation for *P. infestans* strain Rec01. Significant differences from the LB-bacteria free control (Student’s *t* test; *n*=4) are indicated by asterisks (*, *p* < 0.05).
